# High-sensitivity profiling of SARS-CoV-2 noncoding region–host protein interactome reveals the potential regulatory role of negative-sense viral RNA

**DOI:** 10.1128/msystems.00135-23

**Published:** 2023-06-14

**Authors:** Liuyiqi Jiang, Mu Xiao, Qing-Qing Liao, Luqian Zheng, Chunyan Li, Yuemei Liu, Bing Yang, Aiming Ren, Chao Jiang, Xin-Hua Feng

**Affiliations:** 1 Life Sciences Institute, Zhejiang University, Hangzhou, Zhejiang, China; 2 Zhejiang Provincial Key Laboratory of Cancer Molecular Cell Biology, Life Sciences Institute, Zhejiang University, Hangzhou, Zhejiang, China; 3 The MOE Key Laboratory of Biosystems Homeostasis & Protection and Innovation Center for Cell Signaling Network, Life Sciences Institute, Zhejiang University, Hangzhou, Zhejiang, China; Princeton University, Princeton, New Jersey, USA

**Keywords:** SARS-CoV-2, noncoding region RNA, ncrRNA-host protein interactions

## Abstract

A deep understanding of severe acute respiratory syndrome coronavirus 2 (SARS-CoV-2)–host interactions is crucial to developing effective therapeutics and addressing the threat of emerging coronaviruses. The role of noncoding regions of viral RNA (ncrRNAs) has yet to be systematically scrutinized. We developed a method using MS2 affinity purification coupled with liquid chromatography-mass spectrometry and designed a diverse set of bait ncrRNAs to systematically map the interactome of SARS-CoV-2 ncrRNA in Calu-3, Huh7, and HEK293T cells. Integration of the results defined the core ncrRNA–host protein interactomes among cell lines. The 5′ UTR interactome is enriched with proteins in the small nuclear ribonucleoproteins family and is a target for the regulation of viral replication and transcription. The 3′ UTR interactome is enriched with proteins involved in the stress granules and heterogeneous nuclear ribonucleoproteins family. Intriguingly, compared with the positive-sense ncrRNAs, the negative-sense ncrRNAs, especially the negative-sense of 3′ UTR, interacted with a large array of host proteins across all cell lines. These proteins are involved in the regulation of the viral production process, host cell apoptosis, and immune response. Taken together, our study depicts the comprehensive landscape of the SARS-CoV-2 ncrRNA–host protein interactome and unveils the potential regulatory role of the negative-sense ncrRNAs, providing a new perspective on virus–host interactions and the design of future therapeutics. Given the highly conserved nature of UTRs in positive-strand viruses, the regulatory role of negative-sense ncrRNAs should not be exclusive to SARS-CoV-2.

**IMPORTANCE:**

Severe acute respiratory syndrome coronavirus 2 (SARS-CoV-2) causes COVID-19, a pandemic affecting millions of lives. During replication and transcription, noncoding regions of the viral RNA (ncrRNAs) may play an important role in the virus–host interactions. Understanding which and how these ncrRNAs interact with host proteins is crucial for understanding the mechanism of SARS-CoV-2 pathogenesis. We developed the MS2 affinity purification coupled with liquid chromatography-mass spectrometry method and designed a diverse set of ncrRNAs to identify the SARS-CoV-2 ncrRNA interactome comprehensively in different cell lines and found that the 5′ UTR binds to proteins involved in U1 small nuclear ribonucleoprotein, while the 3′ UTR interacts with proteins involved in stress granules and the heterogeneous nuclear ribonucleoprotein family. Interestingly, negative-sense ncrRNAs showed interactions with a large number of diverse host proteins, indicating a crucial role in infection. The results demonstrate that ncrRNAs could serve diverse regulatory functions.

## INTRODUCTION

COVID-19 is an unprecedented global health threat caused by the severe acute respiratory syndrome coronavirus 2 (SARS-CoV-2) (1, 2). As of 31 January 2023, more than 670 million people have been infected, with more than 6.8 million deaths globally (3). As new SARS-CoV-2 variants continue to emerge worldwide, there is an urgent need for a detailed understanding of the molecular determinants of viral pathogenesis. This study will provide new insights into the biology and pathogenic mechanism of SARS-CoV-2 and related coronaviruses and further help to identify potential therapeutic targets.

SARS-CoV-2 is an enveloped, positive-sense, single-stranded RNA virus with a large genome of approximately 30 kb. The genomic structure of SARS-CoV-2 includes 14 open reading frames (ORFs). The largest ORF (ORF1a/b) encodes 16 nonstructural proteins required for the viral RNA productions (4). The remaining ORFs encode nine accessory proteins and four structural proteins: spike (S), envelope (E), membrane (M), and nucleocapsid (N) (5, 6).

**Fig 1:**
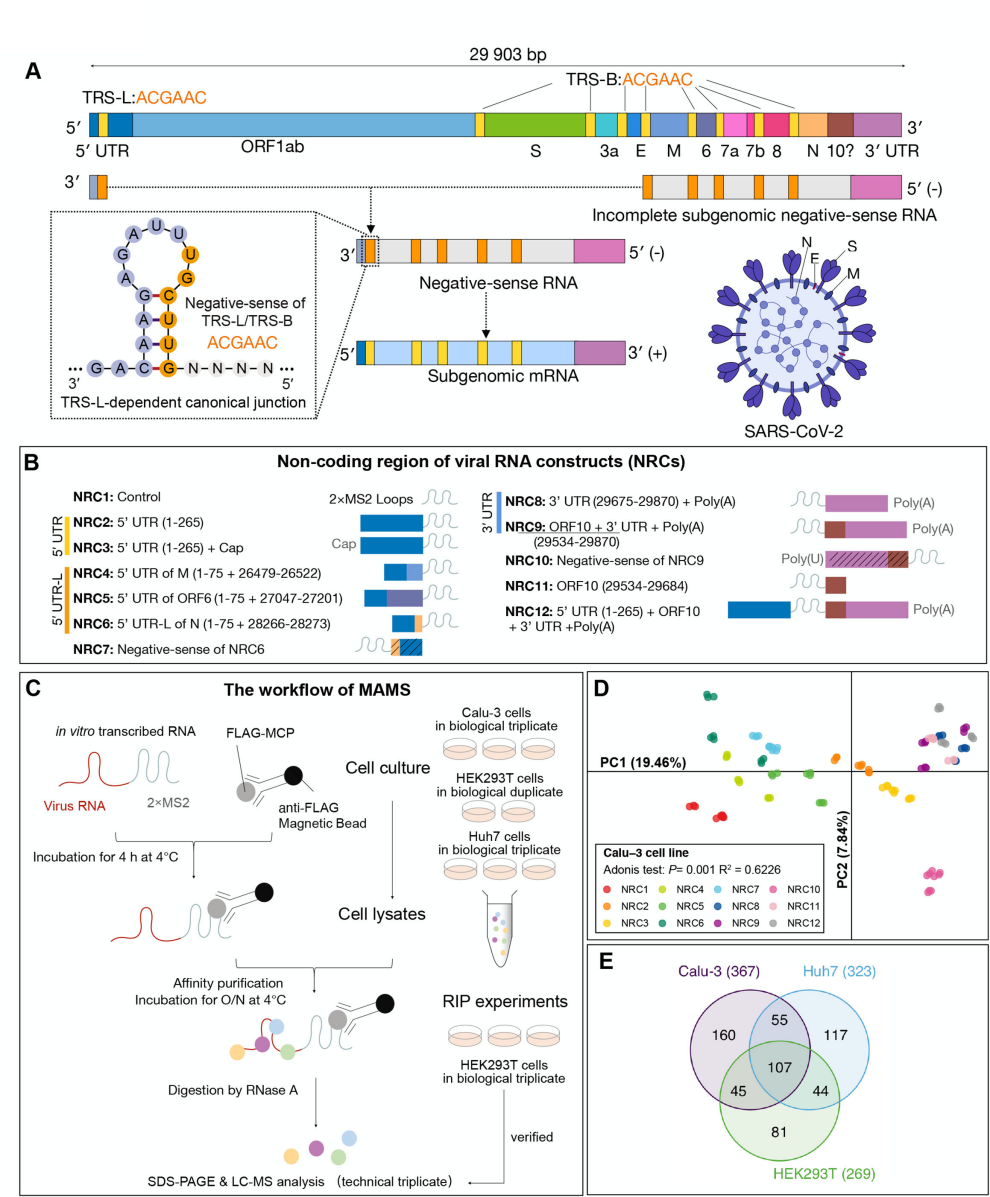
Mapping the SARS-CoV-2 ncrRNA interactome. (**A**) Schematic presentation of SARS-CoV-2 genome (NC_045512) organization, the virion structure, and discontinuous transcription. (**B**) The design of ncrRNA constructs (NRCs). Numbers in parentheses indicate genomic coordinates. (**C**) The workflow of MAMS protocol and verifications. (**D**) Principal component analyses of ncrRNA interactomes in Calu-3. Dots represent different samples. Colors indicate different NRCs. The adonis test was used to determine the statistical significance. (**E**) Venn diagram of SARS-CoV-2 ncrRNA interactomes in Calu-3, Huh7, and HEK239T cells.

Consistent with known RNA viruses, SARS-CoV-2 relies on host proteins for assembling the replication and translation machinery (7). The genomic RNA (gRNA) serves as a dual-purpose template: (i) for the synthesis of the full-length negative-sense RNAs for genome replication and (ii) for the synthesis of diverse subgenomic negative-sense RNAs (−sgRNAs) to make respective subgenomic mRNAs. During transcription, a set of 3′ and 5′ co-terminal −sgRNAs is generated by discontinuous transcription (Fig. 1A) (8, 9). The discontinuous transcription involves a template switch from the body transcription regulatory sequence (TRS-B) to the leader TRS (TRS-L), located at about 70 nucleotides from the 5′ end of the genome (Fig. 1A) (9, 10). To accomplish this, SARS-CoV-2 must employ unique strategies to utilize the host cell proteins while evading the host immune system and defense. Thus, a thorough understanding of the interactions between viral RNAs and host proteins is essential. Several studies have comprehensively characterized SARS-CoV-2 RNA–protein interactome (11 - 15). Schmidt et al. identified physical associations between the viral RNAs and host proteins in infected human cells, revealing key pathways relevant to infection using RNA antisense purification and mass spectrometry (11). By integrating the comprehensive identification of RNA-binding proteins by mass spectrometry (ChIRP-MS) data with genome-wide CRISPR screen data, Flynn et al. demonstrated a physical and functional connection between SARS-CoV-2 RNA and the host mitochondria (12). However, these studies did not systematically investigate the virus–host interactions of the noncoding regions of viral RNA (ncrRNAs).

In this study, we designed a series of MS2-linked viral ncrRNAs and utilized *in vitro* MS2 phage coat protein (MCP) affinity purification to reveal the interacting host proteins via liquid chromatography-mass spectrometry (LC-MS). To identify the core and cell-specific interactome, we performed MAMS in three cell lines: human nonsmall-cell lung cancer cell Calu-3, hepatocellular carcinoma cell Huh7, and transduced human embryonic kidney-derived cell HEK293T. We identified a core SARS-CoV-2 ncrRNA interactome of 58 proteins. We discovered that viral ncrRNAs had complex interactions with host proteins, including pro- and antiviral factors. In particular, the 5′ UTR of viral gRNA was found to interact with host proteins involved in U1 small nuclear ribonucleoprotein (snRNP), while the 3′ UTR had interactions with proteins involved in stress granule (SG) and heterogeneous nuclear ribonucleoprotein (hnRNP) family. Intriguingly, the negative-sense ncrRNAs interacted with an unusually large number and diverse host proteins, which are involved in the regulation of viral production processes, apoptotic signaling, and immune response, indicating an important role of negative-sense RNAs during infection.

## MATERIALS AND METHODS

### RNA purification and preparation

The DNA sequence of each NRC (NRC1-12) was cloned into pUT7 vector bearing the T7 RNA polymerase promoter (16) and was amplified by PCR with *pfu* DNA polymerase, which was used as the templates for transcription. The *in vitro* transcription of all the RNA samples of 12 NRCs was carried out at 37℃ by T7 RNA polymerase, followed by purification with denatured urea-PAGE (polyacrylamide gel electrophoresis) and precipitation with ethanol. The purified RNA samples were annealed at 65℃ for 5 min in the buffer containing 50 mM HEPES (pH 6.8), 50 mM NaCl, and 5 mM MgCl_2_, followed by incubation on ice before pulldown assays.

### *In vitro* RNA capping

NRC3 has the same sequence as NRC2 but with 5′-end 7-methylguanylate cap (cap 0 format). The capping reaction of NRC2 was performed *in vitro* with vaccinia capping enzyme (Yeasen) at 37℃, which catalyzes the addition of 7-methylguanylate structures (cap 0) to the 5′-end of RNA. Phenol-chloroform-isoamyl alcohol extraction was performed to remove the enzyme from the reaction products. After ethanol precipitation and annealing, the capped RNA sample (NRC3) was used for pulldown assays.

### Preparation of 3×FLAG-MCP

The protein sequence was shown as follows, DYKDHDGDYKDHDIDYKDDDDKGGSMASNFTQFVLVDNGGTGDVTVAPSNFANGIAEWISSNSRSQAYKVTCSVRQSSAQNRKYTIKVEVPKGAWRSYLNMELTIPIFATNS DCELIVKAMQGLLKDGNPIPSAIAANSGIY, in which the 3×FLAG-tag was underlined.

For purification, one 6-his-SUMO-tag followed by one ubiquitin-like protease (ULP1) cleavage site was fused to the N-terminal of the 3×FLAG-MCP phage coat protein. The fused protein was expressed in *Escherichia coli* BL21(DE3) Codon plus strain. The lysis of the cultured cells was carried out with a French press in buffer A containing 25 mM Tris-HCl (pH 8.0), 1.0 M NaCl, 5 mM 2-mercaptoethanol, supplemented with 0.1 mM phenylmethylsulfonyl fluoride. After centrifugation, the supernatant sample was loaded onto the first HisTrap column (GE Healthcare) and eluted by buffer B containing 25 mM Tris-HCl (pH 8.0), 500 mM NaCl, 5 mM 2-mercaptoethanol, and 500 mM imidazole. Then the eluted fused protein was incubated with ULP1 protease overnight for cleavage of the fused tag. The cleaved fused 6×His-SUMO-tag was removed from the FLAG-MCP by reloading the sample onto the second HisTrap column (GE Healthcare). The FLAG-MCP was further purified by chromatography using a HiTrap Heparin SP column (GE Healthcare) and a HiLoad Superdex 75 16/60 column (GE Healthcare). The purified FLAG-MCP was concentrated before storage at −80℃ in the buffer containing 40 mM HEPES (pH 7.0), 50 mM KCl, 100 mM NaCl, 5 mM MgCl_2_, and 2 mM dithiothreitol (DTT).

### Cell lysis and digestion

Each sample was separated by SDS-PAGE gel and stained by Coomassie brilliant blue, respectively. Protein gel bands were cut into 1 × 1 × 1 mm^3^ pieces and collected into a 1.5-mL centrifuge tube. Gel pieces were destained with 25% acetonitrile (ACN) in 50 mM ammonium bicarbonate for 20 min at room temperature with shaking. The gel was dehydrated using 1 mL 100% ACN twice with shaking. Proteins were reduced with 10 mM DTT for 60 min at 56℃ and alkylated with 55 mM iodoacetamide for 45 min. Gel pieces were washed with digestion buffer (50 mM NH_4_HCO_3_ [pH 8.0]) twice, dehydrated with acetonitrile, and then dried the sample with speed-vac. Gel pieces were rehydrated with trypsin solution (10 ng/µL sequencing grade modified trypsin, 50 mM NH_4_HCO_3_ [pH 8.0]) and incubated overnight at 37℃. Digested peptides were extracted from gel pieces with elution buffer 1 (50% acetonitrile and 5% formic acid), and elution buffer 2 (75% acetonitrile and 0.1% formic acid) sequentially. Gel pieces were dehydrated with acetonitrile twice, and all supernatants were combined. The peptides solution was dried with speed vac, and digested peptides were resuspended with 5% formic acid and desalted with StageTip.

### RNA pulldown assays

The detailed workflow for RNA pulldown assays in this paper is shown in Fig. 1C. About 50 µL anti-FLAG beads were incubated with excess 3×FLAG-MCP proteins in buffer A (50 mM Tris-HCl [pH7.5], 150 mM NaCl, 0.1% NP-40, and 2 mM MgCl_2_) at 4℃ for 2 h, followed by washing with buffer A twice to remove the unbound proteins. Then the annealed RNA sample was added to the FLAG-MCP bound beads and incubated at 4℃ for 4 h. The unbound RNA was removed by washing with buffer A for three times. In the following step, the cell lysate was added to the RNA-MCP bound beads, and the incubation time was extended to 16 h at 4℃. For each NRC, we used Huh7, Calu-3, and HEK293T cell lines for experiments. After incubation, the cell lysate was removed by washing it five times with buffer A. Then, RNase A was added to digest RNA molecules and release the protein components bound with each RNA sample from the RNA-MCP bound beads. The products were analyzed with SDS-PAGE and followed with liquid chromatography-mass spectrometry or Western blot assay.

### LC-MS data acquisition

Digested peptides were analyzed on a Q Exactive HF-X mass spectrometry system (Thermo Fisher Scientific) equipped with an Easy-nLC 1200 liquid chromatography system (Thermo Fisher Scientific). Samples were injected on a C18 reverse phase column (75 µm × 15 cm, 1.9 µm C18, 5 µm tip). Mobile phase A consisted of 0.1% FA, and mobile phase B consisted of 0.1% FA/80% ACN. Peptides were analyzed with a 60-min linear gradient at a flow rate of 200 nL min^−1^ as the following: 0%–5% B for 2 min, 5%–35% B for 46 min, and 35%–100% B in 12 min. Data-dependent analysis was performed by acquiring a full scan over an *m*/z range of 350–1,500 in the Orbitrap at R=60,000 (*m*/z=200), NCE=27, with a normalized AGC target of 3 × 10^6^, an isolation width of 0.8 *m/z*. The AGC targets and maximum ion injection time for the MS2 scans were 3 × 10^5^ and 60 ms, respectively. Precursors of the +1, +8, or above, or unassigned charge states were rejected; exclusion of isotopes was disabled; dynamic exclusion was set to 45 s. Mass spectrometry data were searched by MaxQuant (version 1.6.10.43).

### Quality control and assessment of LC-MS data

Raw mass spectrometric data files were analyzed by MaxQuant software. All data were searched against the SwissProt Human protein sequences. Peptide and protein identification and label-free quantitation were performed; the false-discovery rate (FDR) was set to 1%; the fixed modification was carbamidomethyl; and the main search peptide tolerance was 10 ppm.

MaxQuant outputs were used for downstream analysis. For each biological replicate, proteins that meet any of the following criteria are filtered out: (i) flagged as potential contaminants; (ii) flagged as reverse sequences; (iii) only identified by site; (iv) quantified by a single razor or unique peptide; (v) only quantified by three or less than three unique peptides; and (vi) only detected once in all samples. Missing data imputation was performed for proteins with missing values in one technical replicate while present in the other two replicates. Imputation was performed using the mean value of the other two technical replicates. Finally, 541, 372, and 312 proteins were detected in Calu-3, Huh7, and HEK293T cells. For data normalization, NormalyzerDE (version 1.5.4) (17) was applied to select the best normalization method. Based on the results of the pooled estimate of variance (PEV), coefficient of variation (CV), median absolute deviation (MAD), and correlation analyses, we chose the variance stabilizing normalization (VSN) as the normalization method (Fig. S2). To remove the batch effects and correct data, we performed the ComBat method using the R package “sva” (version 3.38.0) (18). Principal component analysis (PCA) was applied to examine the batch effects and the separation of interactomes across samples using the R package “ade4” (version 1.7-16) (19). To determine the statistical significances in dissimilarity matrices across samples, the adonis test using the adonis function (999 permutations) in the R package “ade4” (version 1.7-16) (19) was performed. To compare the statistical significances in the dissimilarity of PC1 and PC2, the Kruskal–Wallis test was performed. Pairwise Spearman’s correlation coefficients were calculated for all samples.

### RIP experiment

HEK293T cells were transfected with plasmids co-expressing individual RNA binding protein (RIP; FLAG-tagged), MS2, 5′ UTR, and 3′ UTR. Cells were harvested and resuspended in lysis buffer (50 mM Tris-HCl [pH7.5], 150 mM NaCl, 1% NP-40, and 2 mM MgCl_2_). The cell lysate was incubated with anti-FLAG M2 Magnetic Beads (Sigma-Aldrich, #M8823) at 4℃ for 4 h. After five times washed by lysis buffer, beads were collected. Recovered RNA is isolated by TRIzol. Purified RNAs were detected by quantitative real-time PCR. The relative intensity was calculated as Relative Intensity = Intensity of 5′ UTR (3′ UTR)/Intensity of MS2. Ribbit IgG proteins were used as a negative control.

### Western blot experiment

Western blotting was performed according to standard procedures to validate the interactions between host proteins and NCRs. The protein sample in the SDS loading buffer was denatured at 95°C for 10 min, then separated by SDS-PAGE. After being transformed onto the polyvinylidene difluoride (PVDF) membrane, the protein was immunostained with indicated antibodies and finally detected by horseradish peroxidase-conjugated secondary antibodies and visualized by chemiluminescence (Pierce).

### Nucleotide diversity analysis

To evaluate the degree of conservation of the 5′ UTR and 3′ UTR regions and the other NCRs (ORF10) among the currently known SARS-CoV-2 strains, including Delta and Omicron, we downloaded over 18,000 SARS-CoV-2 genomes that met the length criteria (29,850 bp for the Beta variant; 29,890 bp for other variants) between December 2019 and March 2023 from the NCBI virus database. Due to the limitations of sequencing technology, many genomes have many ambiguous bases, especially in the 5′ UTR and 3′ UTR regions. We only kept the genomes with <1% of ambiguous bases (Fig. S1A). Although all Omicron genomes that qualified from the NCBI virus database were downloaded, only 430 genomes passed quality control. Next, we performed multiple sequence alignment against the SARS-CoV-2 reference genome (NC_045512.2) using MAFFT software (version v7.515) (20). Finally, we calculated the average nucleotide diversity pi (21) of different regions of SARS-CoV-2 to evaluate the conservation.

### Enrichment analyses

Sets of proteins bound to viral ncrRNAs were tested for enrichment of pathways using the Reactome web interface (https://reactome.org/) (22). Statistical significance was set as FDR-adjusted *P* value <0.05. Gene ontology (GO) enrichment analysis was carried out using the R package “clusterProfiler” (version 3.18.0) (23). The GO terms were obtained from the c5 category of the Molecular Signature Database using the R package “msigdb” (version 7.2) (24). Statistical significance was set as *q* value <0.05. Protein domain enrichment analysis was carried out using the STRING web interface (https://string-db.org/) (25) . Statistical significance was set as FDR-adjusted *P* value <0.05.

### RBP validation and site prediction

RBP validation was performed based on the RBP database RBP2GO (26). RBP2GO score is positively correlated with the frequency of the protein being listed as RBP in all data sets. RBP2GO database collected 43 RBP studies in total. RBP site prediction was performed using the RBPsuite (27). The model was set as a specific model. For linear RNAs, RBPsuite predicts the RBP binding scores with them using updated iDeepS (28).

### Protein–protein interaction network analysis

Protein–protein interactions network was performed using the STRING web interface (https://string-db.org/) (25). The minimum required interaction score was set as the highest confidence (0.900). Cytoscape software (version 3.8.2) (29) was applied to visualize the network.

### Weighted gene correlation network analysis

Weighted gene correlation network analysis (WGCNA) analysis was performed in R using the package “WGCNA.” The soft powers were set as 6, 7, and 7 for Calu-3, Huh7, and HEK293T cells, respectively. The network was derived based on signed Spearman correlations. The topological overlap metric was derived from the resulting adjacency matrix and was used to cluster the modules using the blockwiseModules function (blockwiseConsensusModules, for the consensus modules). The dynamic tree cut algorithm was set with a height of 0.25, a deep split level of 2, and a minimum module size of 20. The module-trait correlation *P* values from WGCNA analysis were adjusted by the Bonferroni method.

### Data visualization

Most of the data visualizations were performed in R using the packages “ggplot2” (version 3.3.3) (30), “pheatmap” (version 1.0.12) (31), “ComplexHeatmap” (version 2.6.2) (32), and “UpsetR” (version 1.4.0) (33). The Venn website (http://www.ehbio.com/test/venn/#/) was applied to create a Venn diagram and flower plot.

## RESULTS

### Developing MAMS to map the interactome between SARS-CoV-2 ncrRNAs and host proteins

We developed a method named MS2 affinity purification coupled with liquid chromatography-mass spectrometry to systematically map the interactome between SARS-CoV-2 ncrRNAs and host proteins. We synthesized 12 ncrRNA constructs (NRCs) as baits to investigate the respective host protein interactomes (Fig. 1B). NRC1 comprised 2×MS2 and was designed as a global negative control. NRC2–NRC12 covers different types of ncrRNAs in SARS-CoV-2 (Fig. 1B; Table S1). 5′ UTR and 3′ UTR are the two major ncrRNAs. Previous studies and our nucleotide diversity analysis showed that 5′ UTR and 3′ UTR of SARS-CoV-2 are highly conserved, comparable with viral-coding regions (Fig. S1A and S1B) (34, 35). NRC2 and NRC3 were designed to investigate the interactome of the 5′ UTR; NRC8 and NRC9 were designed to investigate the interactome of the 3′ UTR. SARS-CoV-2 genome has nine TRS cores (5′-ACGAAC-3′), including one TRS-L and eight TRS-Bs; each TRS-B corresponds to an ORF (Fig. 1A; Fig. S1C) (36, 37).

Each subgenomic mRNA joins the TRS-L and the respective TRS-B (Fig. 1A). The distances between TRS-Bs and the start codons of respective ORFs ranged from 0 to 155 bases (Fig. S1C). We selected the TRS-Bs for N, M, and ORF6 as the others are too short. The three TRS-Bs were fused with the leader sequence of 5′ UTR (1–70 nt; referred to as 5′ UTR-L) to construct the complete 5′ UTRs of respective subgenomic mRNAs, namely NRC4, NRC5, and NRC6. The nature of ORF10 in the SARS-CoV-2 genome has been elusive, and data show that ORF10 is not a real protein-coding gene (37 - 39). The low nucleotide diversity (pi value) showed high conservation of ORF10 (Fig. S1B), indicative of its evolutionary importance. We thereby designed the NRC9 and NRC11 to explore the function of ORF10. During the replication and transcription, the full-length and subgenomic negative-sense RNAs are synthesized and serve as templates for progeny viral RNA synthesis and mRNA synthesis, respectively. To investigate the role of negative-sense ncrRNAs, we constructed the reverse complementary of NRC9, namely NRC10, and the reverse complementary of NRC6, namely NRC7. Finally, NRC12 comprising both 5′ UTR and 3′ UTR was synthesized to mimic the full-length viral gRNA excluding the actual ORFs.

All NRCs were prepared by *in vitro* transcription at 37℃ with T7 RNA polymerase, followed by purification with denatured Urea-PAGE and ethanol precipitation (Fig. S1D). 3×FLAG-MCP was expressed as a recombinant protein in *E. coli* BL21(DE3) Codon plus strain and purified with chromatography. The purified 3×FLAG-MCP was bound on anti-FLAG beads for MS2-tagged NRCs RNA binding (see “Materials and methods” for details). The host proteins in cell lysates were incubated with respective NRCs to assemble NRC–protein complexes for affinity purifications. RNase A was applied to release host proteins, which were then identified and quantified by LC-MS (Fig. 1C). To examine the interactomes in different cell line backgrounds, we performed the experiments in human lung cell Calu-3, human liver cell Huh7, and kidney-derived cell HEK293T (40). All experiments were performed with three biological replicates, except for HEK293T cells with two biological replicates. Each biological replicate was then quantified with three technical replicates to ensure the robustness of the approach (Fig. 1C). We implemented data cleaning, data normalization (17), and batch effect correction to address potential biases (18) (Fig. S2; Table S1; see “Materials and methods” for details). These considerations ensured rigorous and statistically meaningful analytical results.

### A comprehensive atlas of proteins bound to the SARS-CoV-2 ncrRNAs

PCA and correlation analysis indicated that the results of experimental and technical replicates are highly concordant (Fig. 1D; Fig. S3A to S3C), indicating the high robustness and sensitivity of the approaches we used. The PCA clustering patterns closely reflected the design strategy in all cell lines (Fig. 1D; Fig. S3B and C). Briefly, samples of NRC2 and NRC3, both of which included 5′ UTR, were more similar; samples of NRC4, NRC5, and NRC6, all of which included 5′ UTR-L, were more similar; and samples of NRC8, NRC9, and NRC11, which included 3′ UTR or ORF10, were more similar. Interestingly, we noticed a clear difference between samples of NRC10 and the other NRCs in Calu-3 and Huh7 cells (Fig. 1D; Fig. S3B).

To delineate the host proteins interacting with ncrRNAs of SARS-CoV-2, we compared NRC2–NRC12 interactomes with the NRC1 interactome to identify statistically enriched proteins against the background (Wilcoxon test; FDR adjusted *P* value <0.05 for Calu-3 and Huh7 cells, FDR adjusted *P* value <0.1 for HEK293T cells). A total of 367 (Calu-3), 323 (Huh7), and 277 (HEK293T) host proteins were identified to interact with the ncrRNAs (Fig. S3D to F; Table S1). Comparing the ncrRNA interactomes of three cell lines revealed that 107 host proteins were conserved (Fig. 1E; Table S1). All 107 host proteins have been identified as human RNA-binding proteins previously (26), confirming their RNA-binding potential (Table S1). We first analyzed the cellular component of these host proteins. GO annotation revealed the strong enrichments of the ribonucleoprotein (RNP) complex and membrane component of cells in all cell lines (Fig. S4A to C). Protein domain enrichment analysis revealed that the SARS-CoV-2 ncrRNA interactomes of all cell lines harbor abundant RNA-binding domains such as RNA recognition motif, DEAD/DEAH box helicase, KH domain, and LSM domain (Fig. S4D to F). These data demonstrated that the MAMS method provided a valuable resource for host proteins interacting with SARS-CoV-2 ncrRNAs. Of note, a previous study showed a depletion of the DEAD/DEAH box helicase domain in the SARS-CoV-2 RNA–host proteins interactome, most likely due to the lack of the 5′ UTR region in the experimental design (14).

### Core SARS-CoV-2 ncrRNA interactomes

**Fig 2:**
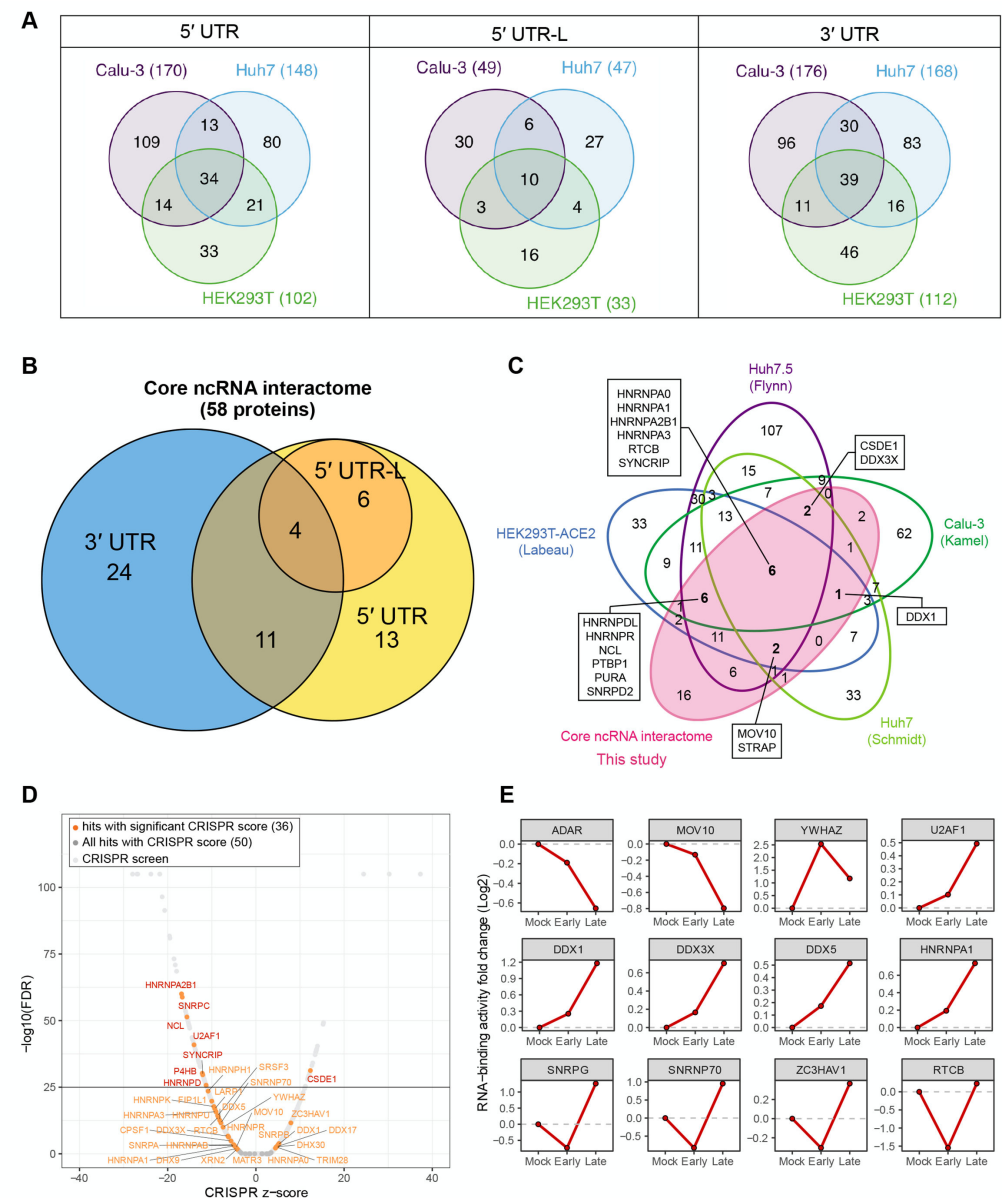
Connecting the SARS-CoV-2 ncrRNA core interactome to perturbations in host cells. (**A**) Venn diagrams of SARS-CoV-2 ncrRNA interactomes of 5′ UTR, 5′ UTR-L, and 3′ UTR in three cell lines. (**B**) Venn diagrams of SARS-CoV-2 ncrRNA core interactomes of 5′ UTR, 5′ UTR-L, and 3′ UTR. (**C**) Intersection of SARS-CoV-2 ncrRNA interactome in this study and RNA interactome from related studies (Flynn et al., (12), Schmidt et al., (11), Kamel et al., (13), and Labeau et al., (15)). (**D**) Volcano plot of proteins in the core SARS-CoV-2 ncrRNA interactome overlaying with published CRISPR screen data in SARS-CoV-2-infected Vero E6 cells. Proteins with highly significant changes (−log_10_FDR >100) were not directly plotted. Orange points denote proteins identified in the core SARS-CoV-2 ncrRNA interactome with significant changes (FDR <0.05 and |CRISPR *z*-score| >2). The red name denotes proteins identified in the SARS-CoV-2 ncrRNA core interactome with highly significant changes (−log_10_FDR>25). (**E**) Fold change of proteins in the core SARS-CoV-2 ncrRNA interactome overlaying with published temporal abundance data of RBP upon SARS-CoV-2 infection. Early represents 8 hpi. Late represents 24 hpi. The RNA-binding activity was measured by quantitative proteomics.

To dissect the biological functions of the viral ncrRNA interactome, we divided NRCs into 5′ UTR, 5′ UTR-L, and 3′ UTR groups based on the design and PCA results (Fig. 1B and D; Fig. S3B and C). To find host proteins enriched in each group, we selected proteins consistently binding to NRCs in the same group in all three cell lines (Fig. 2A; Table S1). Finally, 58 host proteins were identified in all three cell lines, which were defined as the core SARS-CoV-2 ncrRNA interactome (Fig. 2B Table S1). Notably, we observed that all proteins interacting with 5′ UTR-L also interacted with 5′ UTR, as expected (Fig. 2B). To further validate these interactions, we performed RNA Binding Protein Immunoprecipitation experiments in HEK293T (see “Materials and methods” for details). We also performed MS2 affinity purification followed by Western blot analysis to validate some of the key interactions in HEK293T. The results of RIP experiments, Western blot experiments, and mass-spectrometry were highly consistent (Fig. S5A and B) .

We next compared the core ncrRNA interactome with the recently published SARS-CoV-2 RNA interactomes (11 - 15 - 15). We found 72% (42/58) proteins of the SARS-CoV-2 core ncrRNA interactome were enriched in at least one of the studies (Fig. 2C; Table S1). Six proteins were identified in all studies (Fig. 2C) and are involved in mRNA stability and transcriptional regulation (HNRNPA0, HNRNPA1, HNRNPA2B1, HNRNPA3, and SYNCRIP) and a subunit of the transfer RNA-splicing ligase complex (RTCB). To explore the binding specificity of these interactions, we used the RBP binding sites prediction suite (RBPsuite) (27). Among 14 proteins with specifically trained models, 5′ UTR and 3′ UTR had RBP binding sites of 11 proteins, including three known motifs of MATR3, HNRNPA1, and SFPQ (Table S1). These results supported that host proteins interacted with ncrRNAs through sequence-specific binding.

To explore the functional importance of the core interactome, we intersected the members of the core interactome with the published results of a genome-wide CRISPR screen designed to identify host proteins that impact the SARS-CoV-2-induced cell death (12). We obtained CRISPR data for 50 of the 58 proteins in the core ncrRNA interactome, including 36 proteins that significantly impact cell survival after SARS-CoV-2 infection (Fig. 2D). Most proteins may function as antiviral factors. Notably, HNRNPA2B1, SNRPC, NCL, U2AF1, SYNCRIP, P4HB, HNRNPD, and CSDE1 significantly impacted cell death (Fig. 2D). These results highlight the functional importance of the core ncrRNA interactome.

To characterize the dynamics of the core ncrRNA interactome, we intersected our data with the multi-time points (8 and 24 hpi) study of SARS-CoV-2 RNA interactome in Calu-3 cells (13). Intriguingly, most proteins (24/58) in the ncrRNA interactome showed temporal dynamics during the infection (Fig. 2E; Fig. S5C). The RNA-binding activity of several proteins increased throughout the infection, such as DDX1, DDX3X, DDX5, and HNRNPA1, indicating that SARS-CoV-2 requires these proteins in the replication and transcription processes. Several proteins were only upregulated until the late stage, such as SNRPG, SNRNP70, ZC3HAV1, and RTCB. ZC3HAV1 is an antiviral factor; its upregulation possibly hampers SARS-CoV-2 replication. Taken together, these data highlight that the interactions between the ncrRNA and host proteins play essential roles during the replication and transcription processes.

### The 5′ UTR interacts with U1 snRNP and other host factors to promote viral replication and transcription

To characterize the biological functions of the 5′ UTR interactome, we performed GO enrichment analysis on the core interactome of 5′ UTR, revealing the pathways associated with RNA splicing, RNA binding, and ribonucleoprotein complex (Fig. S6A). In eukaryotes, U1, U2, U4, U6, and U5 snRNPs are components of the major spliceosome (41). SNRNP70 and SNRPA are subunits of U1 snRNP. The RIP and MS2 affinity purification showed that SNRNP70 and SNRPA strongly interacted with the 5′ UTR (Fig. S5A and S5B). These results supported the 5′ UTR recruited proteins of U1 snRNP.

**Fig 3:**
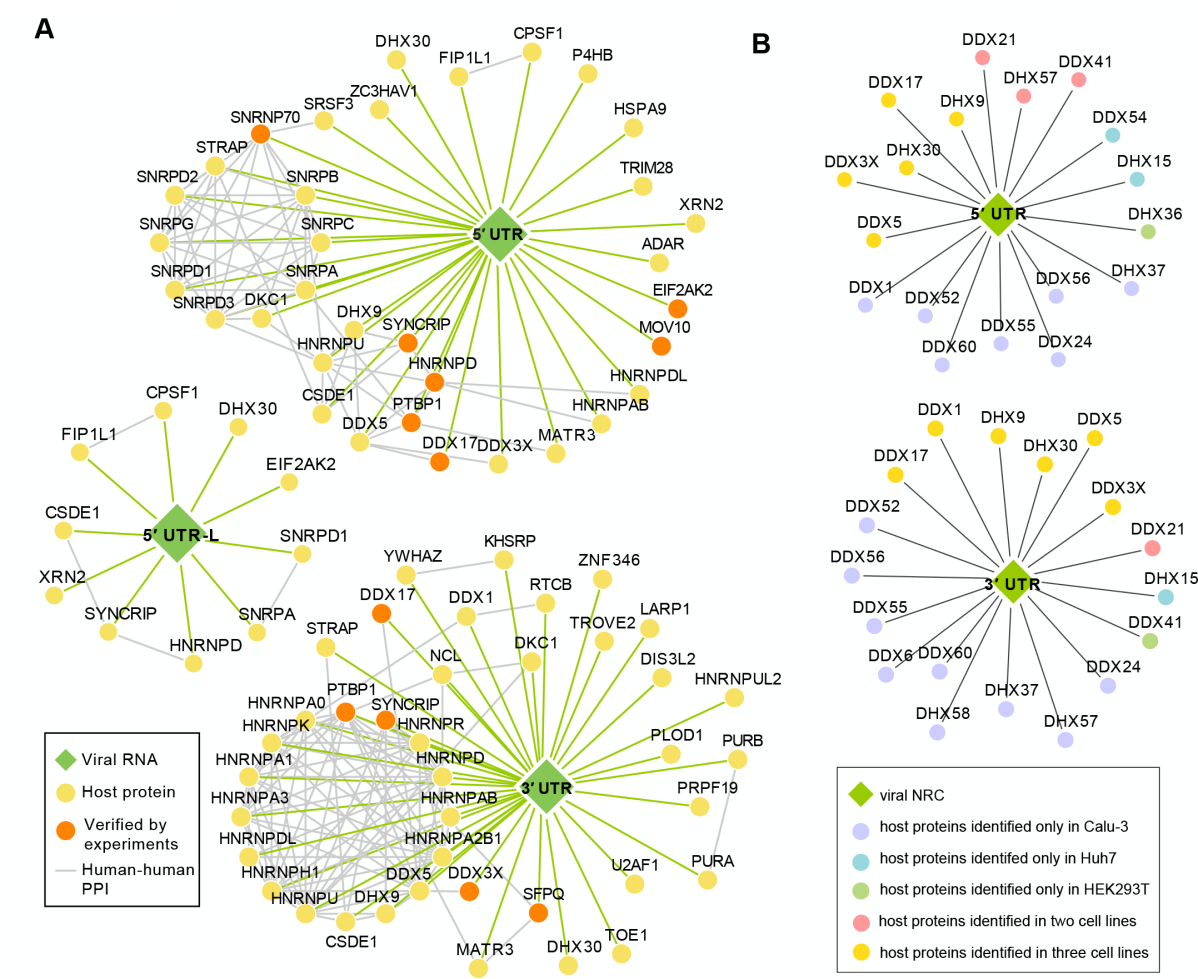
The core SARS-CoV-2 ncrRNA interactome. (**A**) Core interactome of SARS-CoV-2 ncrRNAs. Each node represents a host protein (closed circle) or viral ncrRNA (diamond). Orange circles represent that the interactions between host proteins and viral ncrRNA were verified in the RIP experiments. Green edges indicate the interactions between viral ncrRNAs and host proteins. Gray edges denote the interactions in host proteins. (**B**) Network revealing the interactions between DEAD/DEAH-box helicases and 5′ UTR/3′ UTR. Each node represents a host protein (closed circle) or viral ncrRNA (diamond). Colors are denoted in the box.

To explore the specific interactions between the host proteins and viral 5′ UTR, we visualized the interaction networks using the Cytoscape (Fig. 3A). ADAR, SRSF3, and CSDE1 proteins are potentially pro-viral (Fig. 3A). ADAR is reported to promote RNA virus replication, including SARS-CoV-2 (15, 42). A previous study has also shown that ADAR is involved in SARS-CoV-2 genome editing, a process that may shape the fate of SARS-CoV-2 (43). In HEK293T and A549-ACE2 cells, the knockdown of SRSF3 shows significant inhibition of the SARS-CoV-2 replication. CSDE1 (Unr) is required for Internal Ribosome Entry Site (IRES)-dependent translation in human rhinovirus and poliovirus, which are single-stranded, positive-sense RNA viruses (44, 45). The knockdown of CSDE1 via siRNA also can inhibit the RNA level of SARS-CoV-2 (14). In addition, we noticed the protein P4HA1, an ER-resident prolyl hydroxylase. P4HA1 is reported to be essential for the HIF-1α stabilization (46), which is activated when SARS-CoV-2 attacks the lung and impairs gas exchange leading to systemic hypoxia (47). The mass-spec and RIP data revealed that the protein P4HA1 interacted with 5′ UTR in all cell lines (Fig. S5A and S6B). Knockdown of P4HA1 and P4HA2 led to strong inhibition of Zika and Dengue virus (48); thereby, the P4HA1 may play a similar role for SARS-CoV-2. Taken together, these proteins may promote viral entry, transcription, and replication by interacting with 5′ UTR.

From the host side, antiviral factors ZC3HAV1, MOV10, EIF2AK2, and TRIM28 (49 - 54) had interactions with 5′ UTR (Fig. 3A). ZC3HAV1 (also named ZAP) has been shown to act as an inhibitor of SARS-CoV-2 programmed ribosomal frameshifting to disturb the translation of the viral RNA (49). A previous study reported that MOV10 could enhance IFN response using an RIG-I-like receptor (RLR)-independent pathway to provide antiviral activity against SARS-CoV-2 in the same way (52). EIF2AK2 (also named PKR), an interferon-induced serine-threonine protein kinase, can prevent viral replication by phosphorylating the initiation factor eIF2α (55). Knockdown of TRIM28 is reported to promote SARS-CoV-2 cell entry and replication (15, 56). Thus, these proteins may interact with 5′ UTR to restrict SARS-CoV-2, indicating that the host may use the 5′ UTR to control viral cell entry, transcription, and replication.

### The 3′ UTR interactome is involved in stress response

For SARS-CoV-2 and all coronaviruses, viral mRNAs and gRNAs share the same 3′ UTR. GO enrichment analysis showed that the 3′ UTR interactome is enriched with pathways associated with RNA splicing, RNA binding, and cytoplasmic SG (Fig. S6C). A strong interplay is previously detected between SARS-CoV-2 RNA and stress granules. SGs can exert antiviral functions via various mechanisms, sequestrating host and viral mRNAs and proteins, recruiting immune signaling intermediates, and inhibiting protein synthesis (57 - 59). G3BP1 is crucial to forming SGs (60); although our mass-spec data did not show interactions between G3BP1 and the 3′ UTR, our RIP and MS2 affinity purification experiments confirmed that G3BP1 binds to 3′ UTR in HEK293 cells (Fig. S5A and B). On the host side, G3BP1 inhibits SARS-CoV-2 by promoting SG formation. From the viral side, the SARS-CoV-2 N protein sequesters the G3BP1 to decrease the SG formation (61, 62). These observations demonstrate that the 3′ UTR is important to the SG localization of SARS-CoV-2.

Next, we explored the interactions between 3′ UTR and host proteins in detail. Several proteins of the hnRNP family interacted with 3′ UTR (Fig. 3A). The hnRNPs are the major factors responsible for RNA processing, including RNA splicing, maturation, decay, and translation (63). Recent articles show that hnRNPs play diverse roles during SARS-CoV-2 infection. For example, to facilitate viral RNA processing, SARS-CoV-2 hijacks host HNRNPA2B1 protein redistribution through direct binding with NSP1 to impair the host innate immunity (64). HNRNPK may also be a pro-viral factor. The drug phenethyl isothiocyanate targeting HNRNPK shows a significant inhibitory effect on SARS-CoV-2 (15). HNRNPA1 has been reported to regulate early translation to replication switch in SARS-COV-2 (65). Thus, the interplays between the hnRNPs and SARS-CoV-2 may assist the viral replication and response to the host immune regulations. On the other hand, the host proteins may target viral 3′ UTR to inhibit viral replication. LARP1 was identified in the 3′ UTR interactome (Fig. 3A), consistent with a previous report using the Vero cells (11). In HEK293 cells, the knockout of LARP1 promotes the production of SARS-CoV-2 RNA (11), suggesting that LARP1 can repress viral replication. Taken together, these results indicate that the interactions between 3′ UTR and host proteins have facilitated the replication and infection of SARS-CoV-2 while localizing the viruses to the SG.

### The 5′ UTR and 3′ UTR interact with diverse helicases

Helicases are essential for viral genome replication, transcription, and translation (66). We discovered that the 5′ UTR and 3′ UTR interacted with an extensive array of DEAD/DEAH-box helicases in at least two cell lines (Fig. 3B), including DDX3X, DDX5, DDX17, DDX21, DHX9, and DHX30. 5′ UTR-L also interacted with several DEAD/DDX-box helicases (Fig. S6D). Among these helicases, DDX3X, DDX1, DDX5, and DDX6 are reportedly hijacked by several RNA viruses to facilitate various steps of their replication cycles (67 - 70). A study shows that DDX3X colocalizes with SARS-CoV-2 RNA foci, and its inhibition significantly reduces the viral replication (71). DDX1, DDX5, and DDX6 RNA helicases are required for the SARS-CoV-2 replication (70). Notably, a previous study has shown that the specific interaction of DDX6 with an RNA hairpin in the 3′ UTR of the Dengue virus genome mediates G1 phase arrest, indicating the SARS-CoV-2 utilizes a similar strategy to alter host cell fates (72). In contrast, DDX17, the paralogs of DDX5, may have an antiviral function as a cofactor for ZC3HAV1 to promote the viral RNA decay (73). DDX21 is also an antiviral factor. A previous study reveals that DDX21 interacts with the 5′ UTR of the Borna disease virus (BDV) X/P polycistronic mRNA to inhibit its translation. *In vitro*, RNA folding assays suggest that DDX21 binding causes structural alterations in the 5′ UTR of the BDV mRNA, thus interfering with the reinitiation of translation by ribosomes on this polycistronic message (74). Intriguingly, DDX21 has been reported to have a strong inhibition of the SARS-CoV-2 infection (70), indicating DDX21 may modify the SARS-CoV-2 5′ UTR structure. These results show that DDX17 and DDX21 may interact with 5′ UTR and 3′ UTR to inhibit the SARS-CoV-2 translation. Taken together, DEAD/DEAH-box helicases might play regulatory roles during viral replication and translation via interactions with 5′ UTR and 3′ UTR.

### WGCNA revealed NRC-related functional modules

**Fig 4:**
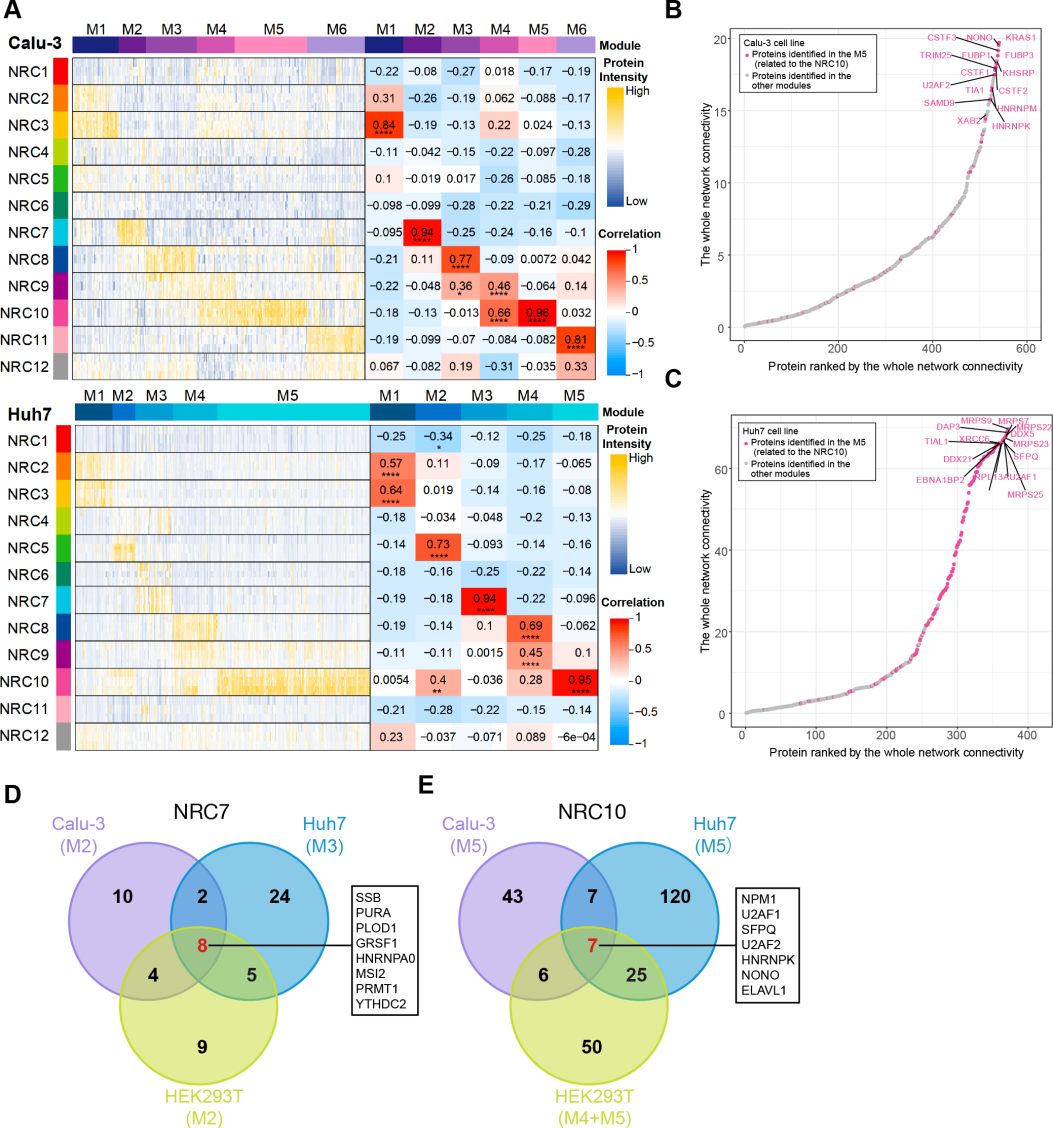
Modules of proteins strongly associated with different ncrRNA were identified by weighted gene coexpression network analysis. (**A**) Heatmap and table showing that modules were strongly correlated with different ncrRNAs in Calu-3 (top) and Huh7 (bottom). Heatmap shows the intensity pattern of host proteins in different ncrRNA interactomes, as characterized into different modules. The table shows the correlations between different modules and NRCs. *P* values were adjusted by the Bonferroni method. *P < 0.05, **P < 0.01, ***P < 0.001, ****P < 0.0001. (**B, C**) Plots showing that the proteins identified in the modules correlated with the NRC10 had the highest connectivity in the whole network in Calu-3 (**D**) and Huh7 (**E**) cells. The top 15 proteins in the modules were highlighted in color pink. (**D**) Venn diagram of NRC7-related modules in Calu-3, Huh7, and HEK293T cells. (**E**) Venn diagram of NRC10-related modules in Calu-3, Huh7, and HEK293T cells.

Weighted gene correlation network analysis is a powerful method to uncover *de novo* regulatory modules without making prior assumptions (75). The abundances of 367, 323, and 269 proteins were used for WGCNA for the Calu-3, Huh7, and HEK293T, respectively, leading to the identification of 10, 6, and 7 modules for each cell line. The significance of correlations between modules and NRCs was calculated. We focused on the modules that had significant positive correlations with NRC2–NRC12; six, five, and five modules for the Calu-3, Huh7, and HEK293T were identified, respectively (Fig. 4; Fig. S7A; Table S1). Strikingly, the filtered modules closely reflected the grouping of the NRCs, indicating that the grouping of NRCs, although subjective, reflects the true biology of the ncrRNA–host protein interactions (Fig. 4A; Fig. S7A). Briefly, module 1s (M1s) had strong positive correlations with both NRC2 (5′ UTR) and NRC3 (5′ UTR + Cap) in all three cell lines; M3 in Calu-3 and M4 in Huh7 showed positive correlations with both NRC8 (3′ UTR) and NRC9 (ORF10 + 3′ UTR) (Fig. 4A; Fig. S7A). Notably, M2, M4, and M5 for Calu-3; M3 and M5 for Huh7; M2, M4, and M5 for HEK293T strongly correlated with the two negative-sense ncrRNAs, indicating the unique role of negative-sense ncrRNAs. Moreover, except for the HEK293T cell line, we found that almost all proteins identified in the modules related to the NRC10 showed the highest or relatively higher connectivity in the *de novo* interacting network in the three cell lines (Fig. 4B and C;Fig. S7B), further suggesting the importance of the negative-sense ncrRNA interactomes.

To confirm our previous findings of the core interactome, we explored the functions of modules related to 5′ UTR and 3′ UTR. Comparing the M1s in different cell lines, 13 proteins were shared, and most of them (12/13) were also identified in the 5′ UTR core interactome (Fig. S7C; Table S1). The proteins enriched in the M1s were involved in RNA splicing and U1snRNP (Fig. S7D), consistent with the 5′ UTR core interactome analysis. For the modules associated with 3′ UTR, 8 of 11 proteins shared in all cell lines were also identified in the 3′ UTR core interactome (Fig. S7E; Table S1). The GO enrichment analysis also revealed the enrichment of proteins involved in the mRNA 3′ UTR binding and SG (Fig. S7F), consistent with our analysis of the 3′ UTR core interactome. Therefore, hypothesis-free WGCNA is highly consistent with the hypothesis-driven core-interactome analysis, further supporting the important roles of 5′ UTR and 3′ UTR interactomes in virus–host interactions.

### The negative-sense ncrRNAs play regulatory roles in virus–host interactions

The negative-sense viral RNAs are known to serve as the templates for replication and transcription. However, the regulatory roles of negative-sense RNAs in viral infection remain unexplored. We next investigated the modules related to negative-sense ncRNAs. The NRC7 is the negative-sense of NRC6 (5′ UTR-L of N or the 5′ UTR of the subgenomic mRNA of N gene), which is the negative-sense of 5′ UTR -L sequence plus eight bases. Although NRC7 is relatively short, eight proteins (SSB, HNRNPA0, PURA, MSI2, PLOD1, PRMT1, GRSF1, and YTHDC2) were identified to interact with the NRC7 in all cell lines (Fig. 4D; Table S1). Among these proteins, SSB (La protein), GRSF1, and MSI2 may be hijacked by SARS-CoV-2 to promote translation and disturb the host immune response. Pioneering studies have shown that the cellular SSB shields nonsegmented negative-strand RNA viral leader RNA from RIG-I and enhances virus growth by multiple mechanisms (76). GRSF1 has been shown to stimulate the translation of viral mRNAs, such as the influenza virus (77). MSI2 is a member of the Musashi protein family, which is reported to be downregulated in the SARS-CoV infection (78). A drug targeting MSI2 has shown a moderate inhibiting effect of the SARS-CoV-2 (13). PRMT1 is a host protein arginine methyltransferase (PRMT). SARS-CoV-2 proteins are known to be methylated by PRMTs (79). YTHDF2 is an m^6^A reader (80), and m^6^A is widely distributed in both positive-sense and negative-sense SARS-CoV-2 RNA (81). Therefore, the methylation of NRC7 may regulate the viral transcription and replication through YTHDF2. Taken together, these results suggest that NRC7 plays an important role in SARS-CoV-2 transcription and replication processes.

We next analyzed the modules associated with the NRC10, the negative-sense of NRC9 (ORF10 + 3′ UTR). Consistent with the PCA results, the results of WGCNA revealed the unusual role of NRC10, which had specific interactions with many host proteins in all three cell lines (Fig. 4A; Fig. S7A). Seven proteins (NPM1, U2AF1, U2AF2, SFPQ, NONO, ELAVL1, and HNRNPK) were identified in modules specifically associated with the NRC10 in three cell lines (Fig. 4E; Table S1). ELAVL1, NPM1, and SFPQ may act as pro-viral factors. ELAVL1 is a known stabilizer of RNA. A recent study shows that the ELAVL1-inhibiting drug can suppress the SARS-CoV-2 protein production, indicating the pro-viral role of ELAVL1 (13). The nucleophosmin1 (NPM1, also named B23) is involved in several cellular processes. Overexpression of NPM1 promoted the porcine epidemic diarrhea virus (PEDV), a member of coronavirus, while knockdown of NPM1 suppressed PEDV growth (82). SFPQ is a member of the *Drosophila behavior/human splicing* (DBHS) protein family. Studies have shown that SFPQ is a pro-viral host factor essential for virus replication and transcription, including encephalomyocarditis virus (EMCV), influenza A virus, and Epstein–Barr virus (83 - 85). Another member of the DBHS protein family, NONO, may play the opposite function. NONO has been identified to display antiviral activity during SARS-CoV-2 infection via CRISPR screens (12). These results showed that the interactions between these proteins and NRC10 could potentially regulate the SARS-CoV-2 replication and transcription.

**Fig 5:**
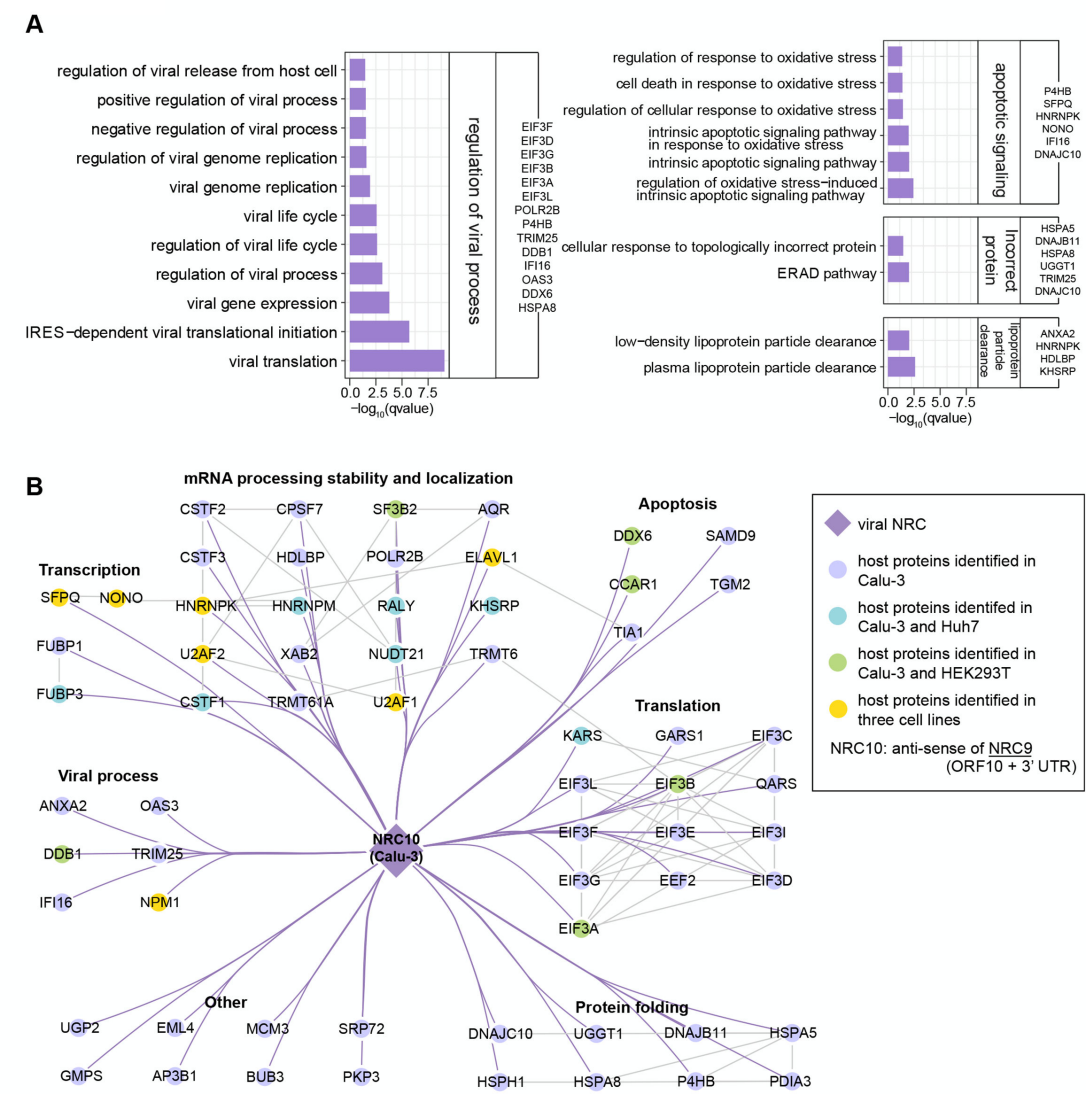
Negative-sense ncrRNAs interact with host proteins involved in the regulation of viral processes. (**A**) GO enrichment results revealing the major functions of NRC10-related modules in Calu-3. (**B**) Network showing the interactome of NRC10 in different cell lines. Each node represents a host protein (closed circle) or viral ncrRNA (diamond). Purple edges indicate the interactions between viral ncrRNAs and host proteins. Gray edges denote the interactions between host proteins. Colors are denoted in the box.

When infected, different cell lines exhibited drastically different phenotypes; we performed the GO enrichment of NRC10 interactomes for each cell line to identify the cell-line-specific features (Fig. 5A; Fig. S7G and H). In the Calu-3 cell line, NRC10 had a significant number of interactions with the subunits of the eukaryotic translation initiation factors 3 (eIF3) complex and proteins involved in response to the incorrect protein and oxidative stress (Fig. 5A and B). SARS-CoV-2 nsp1 has been reported to be adjacent to subunits of the eIF3 complex to inhibit host translation in the human nonsmall cell lung carcinoma cell line H1299 (86). In the Huh7 cells, we discovered that the NRC10 had strong interactions with ribosomal proteins in the cytoplasm and mitochondria (Fig. S7G; Fig. S8A). Liver cells are enriched with mitochondria, which are needed for hepatic metabolism. In the event of an infection, mitochondria contribute to immunity by multiple mechanisms (87, 88) and are the targets of diverse positive-sense single-stranded RNA viruses, including SARS-CoV-2 (89, 90). Therefore, NRC10 may recruit these proteins to disturb immunity via mitochondria to facilitate its replication. Moreover, the NRC10 interactomes of Huh7 and HEK293T were enriched in the proteins involved in the innate immune response (Fig. S7G and H; Fig. S8), which provided a potential explanation for the different cell death ratios after the infection of the SARS-CoV-2 (40). Taken together, these data demonstrated that the negative-sense ncrRNAs, in addition to serving as direct templates for transcription and replication, may also have diverse regulatory roles in viral protein folding, translation, transcription, host immune response, and mitochondria dynamics.

## DISCUSSION

**Fig 6:**
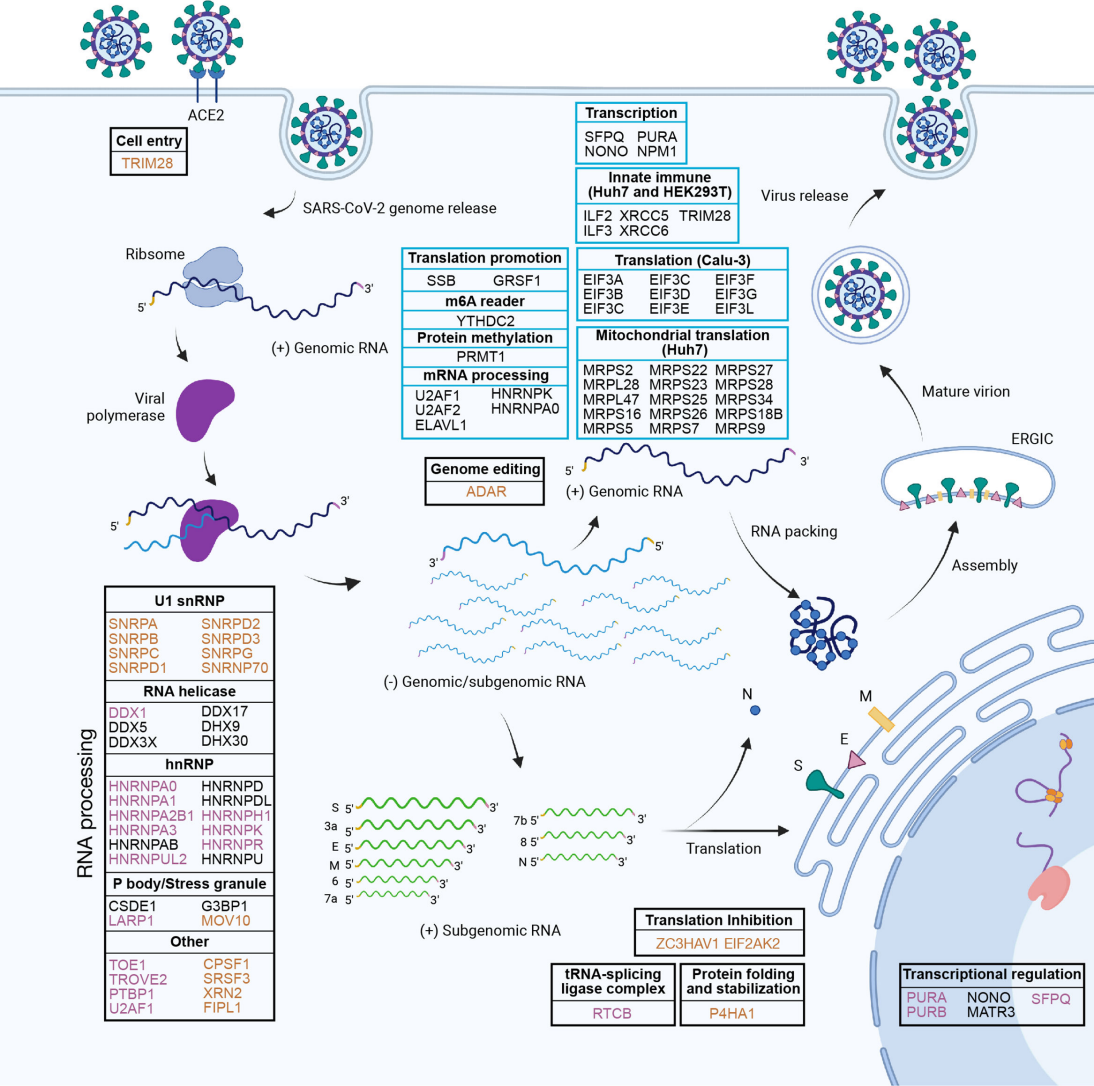
Summary of the functions of SARS-CoV-2 ncrRNA interactomes. Host proteins in the black boxes interacted with positive-sense ncrRNAs (5′ UTR and 3′ UTR). Host proteins in the blue boxes interacted with negative-sense ncrRNAs. Brown color denotes proteins that interacted with 5′ UTR specifically. Purple color denotes proteins that interacted with 3′ UTR specifically.

Decoding how the ncrRNAs of SARS-CoV-2 interact with host proteins is crucial to understanding virus biology and COVID-19. We developed the MAMS method to systematically map the viral ncrRNA–host protein interactome across three cell lines with high sensitivity. Compared with previous studies, the MAMS approach removed the potential masking effects exerted by the high abundance of viral proteins during *in vivo* infection and revealed significantly more host proteins binding with the viral ncrRNAs. Integration of the MAMS data from the three cell lines with RIP data revealed the important role of core SARS-CoV-2 ncrRNA interactome and the unique role of negative-sense ncrRNAs (Fig. 6).

In particular, the viral 5′ UTR interacted with P4HA1 and proteins of U1 snRNP to assist its viral replication and transcription. These proteins are potential drug targets. A previous study reveals that the viral NSP16 protein can bind to the recognition domain of U1/U2 snRNAs to disrupt the host mRNA splicing (91). Thus, our findings provide a new mechanism through which snRNPs assist the transcription and replication of SARS-CoV-2. On the contrary, antiviral factors ZC3HAV1, MOV10, and EIF2AK2 may interact with 5′ UTR to inhibit viral replication and transcription.

On the other hand, viral 3′ UTR had strong associations with proteins related to SGs and the hnRNP family. SARS-CoV-2 modulates the host proteins to inhibit the antiviral SGs formation and maximize the viral replication efficiency (91, 92). For example, N protein-mediated SG disassembly can promote the production of SARS-CoV-2 (91). Interactions between 3′ UTR and SGs may play an important role in the transcription and replication of SARS-CoV-2. Conversely, the hnRNP family could play a pro-viral function via nucleic acid metabolism to promote viral replication and transcription.

The function of ORF10 remains to be determined. Originally, SARS-CoV-2 genome annotation positioned ORF10 upstream of 3′ UTR. However, evidence suggests that the ORF10 is not at a protein-coding region. Katarzyna et al. found that the replication of a SARS-CoV-2 variant in which the ORF10 gene was prematurely terminated was not impacted *in vitro* or *in vivo* (39). The presence of the subgenomic mRNAs corresponding to the ORF10 is also questionable. Jungreis et al. analyzed the SARS-CoV-2 genome by comparing 44 *Sarbecovirus* genomes and showed that while the sequences are highly conserved, premature stop-codons can be observed in all but the SARS-CoV-2-related genomes (93). The PCA analysis showed no significant separation among the NRC8 (3′ UTR), NRC9 (ORF10 + 3′ UTR), and NRC11 (ORF10) (Fig. 1D; Fig. S3B and C). However, NRC10, the negative-sense of NRC9, which also includes ORF10, was demonstrated to be distinct from the group (Fig. 1D; Fig. S3B). The analysis of the NRC10-related modules revealed that some proteins involved in regulating the viral process, mitochondrial translation, ERAD pathway, and immune response show cell-line specificity. Intriguingly, recent studies reveal the relationship between disrupted cardiac mitochondria postinfection and cardiovascular dysfunction in COVID-19 patients (94). These results indicate that the negative-sense, instead of the positive-sense or the protein product of ORF10, may have important functional roles.

Besides NRC10, NRC7, the negative-sense of NRC6 (5′ UTR-L of N), was also of interest. NRC7 is almost equivalent to the negative-sense of 5′ UTR-L (Fig. 1B). NRC7 interacted with a series of host proteins. Eight proteins were identified to interact with NRC7 in all cell lines, including SSB. A previous study has shown that the SSB interacts with nonsegmented negative-strand RNA viral leader RNA to promote virus replication. Thus, the SSB may have a similar function in SARS-CoV-2 (76). We consider the SSB as a new potential target to inhibit the infection of SARS-CoV-2.

For most positive-strand RNA viruses, the negative-sense RNAs serve as the templates for synthesizing positive-sense gRNA and subgenomic RNAs. Interestingly, a study found that negative-sense RNAs are heavily modified by m^6^A in the 5′ UTR and many coding regions, while the positive-sense RNAs are heavily modified by m^6^A only in the 3′ UTR, indicating that negative-sense RNAs are methylated differently *in vivo* (81). In a separate study, the negative-sense coronaviral RNA was found to be functionally relevant to the activation of host innate immune responses through the cleavage of their 5′-end polyuridine (polyU) sequences, a process mediated by the highly conserved viral endoribonuclease nsp15 (95). Our study demonstrates the strong potential of diverse regulatory roles of the negative-sense viral ncrRNAs in the entire process of viral infection. Given the highly conserved nature of UTRs in coronaviruses and more distantly related positive-strand RNA viruses, it is tempting to infer that the negative-sense RNAs in diverse positive-strand RNA viruses, such as Zika and Dengue viruses, likely have similar regulatory roles in virus–host interactions.

In summary, our work reveals the comprehensive and highly sensitive landscape of the functional SARS-CoV-2 ncrRNA interactome in diverse cell lines. More importantly, integrated analyses show that the negative-sense viral RNAs could play previously underappreciated regulatory roles in various virus–host interactions. These findings introduced a new mechanistic perspective on how SARS-Cov-2, and possibly other positive-strand RNA viruses, utilizes the negative-sense viral RNAs to manipulate and interact with host cells, providing a new avenue of antiviral drug discovery.

### Limitations of the study

Although this study provides a rich resource of SARS-CoV-2 noncoding region–host protein interactome, several limitations exist due to the restricted access to a P3 lab to perform the infection experiments. First, the interactions between ncrRNAs and host proteins were identified based on the whole cell lysate from uninfected cells. Therefore, our findings may not fully represent the complexity of a real infection, and some interactions identified may be false positives. Although our *in vitro* assays demonstrated potential interactions between these proteins and the NCRs, the subcellular distribution of these proteins could prevent the interactions *in vivo*. Second, this study needed more functional validations to reveal the potential biological significance of the identified interactions in an infection context.

## Data Availability

The mass-spec data after normalization for all experiments is provided in this paper. The source code is available on GitHub (https://github.com/jlyq617/SARS-CoV-2).

## SUPPLEMENTAL MATERIAL

Fig. S1 msystems.00135-23-s0001.pdf Design and validation of NRCs. (A) SARS-CoV-2 sequence information, as downloaded from the NCBI Virus database. (B) Average nucleotide diversity (pi value) of different regions of SARS-CoV-2. (C) Position information of TRS-L/TRS-Bs in the SARS-CoV-2 genome. (D) Gel graphs of 12 NRCs after purification.

Fig. S2 msystems.00135-23-s0002.pdf Systematic evaluation of different normalization methods. (A) Bar plots demonstrating the PCV (left), PMAD (middle), and PEV (right) statistics of different normalization methods when compared with the log2 transformation in Calu-3 cells (the lower the better). (B) Box plots showing the Pearson correlation (left) and Spearman correlation (right) between raw data and normalized data in Calu-3 cells. (C) Bar plots demonstrating the PCV (left), PMAD (middle), and PEV (right) statistics of different normalization methods compared with the standard log2 transformation in Huh7 cells. (D) Box plots showing the Pearson correlation (left) and Spearman correlation (right) between raw data and normalized data in Huh7 cells. (E) Bar plots demonstrating the PCV (left), PMAD (middle), and PEV (right) statistics of different normalization methods when compared with the log2 transformation in HEK293T cells (The lower the better). (F) Box plots showing the Pearson correlation (left) and Spearman correlation (right) between raw data and normalized data in HEK293T cells.

Fig. S3 msystems.00135-23-s0003.pdf Overview of the ncrRNA interactomes in cell lines. (A) Heatmaps of the interactomes revealing that the biological and technical replicates of NRCs are highly correlated in Calu-3 (left), Huh7 (middle), and HEK293T (right) cells. (B, C) PCA analyses show ncrRNA interactomes’ patterns in Huh7 (B) and HEK293T (C). Dots represent different samples. Colors indicate different NRCs. The adonis test was used to determine statistical significance. (D–F) Overview of the numbers of host proteins bound to different NRCs in Calu-3 (D), Huh7 (E), and HEK293T (F) cells. See also Table S1.

Fig. S4 msystems.00135-23-s0004.tif Cell components and domain analysis of SARS-CoV-2 ncrRNA interactome. (A–C) Gene ontology cellular component analysis of host proteins enriched in SARS-CoV-2 ncrRNA interactomes in Calu-3 (A), Huh7 (B), and HEK293T (C) cells. (D–F) Protein domain enrichment analyses of host proteins enriched in SARS-CoV-2 ncrRNA interactomes in Calu-3 (D), Huh7 (E), and HEK293T (F) cells.

Fig. S5 msystems.00135-23-s0005.pdf Functional characterizations of the core SARS-CoV-2 ncrRNA interactome. (A) RIP experiments validating the interactions between host proteins and SARS-CoV-2 5*'* UTR or 3*'* UTR. P values were calculated by the Wilcoxon test. **P*< 0.05, ***P* < 0.01, ****P* < 0.001. Ribbit IgG proteins (rIgG) were used as a negative control. (B) Western blot experiments validating the interactions between host proteins and SARS-CoV-2 5*'* UTR or 3*'* UTR. (C) Fold change of proteins in the core SARS-CoV-2 ncrRNA interactome overlaying with published temporal abundance data of RBP during SARS-CoV-2 infection. Early represents 8 hpi.

Fig. S6 msystems.00135-23-s0006.pdf The interactome of SARS-CoV-2 5*'* UTR, 3*'* UTR, and 5*'* UTR-L. (A) GO enrichment results revealing the major functions of the 5*'* UTR interactome. (B) The boxplot showing the intensity of P4HA1 in Calu-3 and Huh7 cells. *P* values were calculated by the Wilcoxon test. **P* < 0.05, ***P* < 0.01, ****P* < 0.001. (C) GO enrichment results revealing the major functions of the 3*'* UTR interactome. (D) Network revealing the interactions between DEAD/DEAH-box helicases and 5*'* UTR-L. Each node represents a host protein (closed circle) or viral ncrRNA (diamond). Colors are denoted in the box.

Fig. S7 msystems.00135-23-s0007.pdf ncrRNA-related modules identified by WGCNA. (A) Heatmap and table showing that modules were strongly related to different NRCs based on the interactions between host proteins and NRCs in the HEK293T cell line. Heatmap shows the intensity patterns of host proteins in different ncrRNA interactomes. The table shows the correlations between different modules and NRCs. *P* values were adjusted by the Bonferroni method. **P* < 0.05, ***P* < 0.01, ****P* < 0.001, *****P* < 0.0001. See also Table S1. (B) Plots showing that the whole network connectivity of proteins identified in the modules correlated with the NRC10 in HEK293T cells. The top 15 proteins were highlighted in color pink. (C) Venn diagram of 5*'* UTR-related modules in Calu-3, Huh7, and HEK293T cells. (D) GO enrichment results reveal the major functions of the 5*'* UTR-related modules. (E) Venn diagram of 3*'* UTR-related modules in Calu-3, Huh7, and HEK293T cells. (F) GO enrichment results reveal the major functions of the 3*'* UTR-related modules. (G) GO enrichment analysis of NRC10 interactomes in Huh7 cells. (H) GO enrichment analysis of NRC10 interactomes in HEK293T cells.

Fig. S8 msystems.00135-23-s0008.pdf Networks of NRC10 interactomes in Huh7 (A) and HEK293T (B) cells. Each node represents a host protein (closed circle) or viral ncrRNA (diamond). Blue or green edges indicate the interactions between viral ncrRNAs and host proteins. Gray edges denote the interactions in host proteins. Colors are denoted in the box.

TABLE S1 msystems.00135-23-s0009.xlsx Profiling of SARS-CoV-2 noncoding region–host protein interactome in Calu-3, Huh7, and HEK293T cells: 12 NRCs for RNA pulldown assays (sheet 1); full data for all proteins in all datasets including proteomes of Calu-3, Huh7, and HEK293T, related to Fig. 1D (sheets 2–4); the enrichment of NRC2-12 over NRC1, and the overlaps of SARS-CoV-2 ncrRNA interactome in three cell lines, related to Fig. 1E and Fig. S3 (sheets 5–9); the core SARS-CoV-2 ncrRNA interactome, related to Fig. 2 and Fig. 3 (sheets 10–12); The modules identified by WGCNA, related to Fig. 4 and Fig. S7 (sheets 13–14)

ASM does not own the copyrights to Supplemental Material that may be linked to, or accessed through, an article. The authors have granted ASM a non-exclusive, world-wide license to publish the Supplemental Material files. Please contact the corresponding author directly for reuse.

## References

[1] Guan W-J , Ni Z-Y , Hu Y , Liang W-H , Ou C-Q , He J-X , Liu L , Shan H , Lei C-L , Hui DSC , Du B , Li L-J , Zeng G , Yuen K-Y , Chen R-C , Tang C-L , Wang T , Chen P-Y , Xiang J , Li S-Y , Wang J-L , Liang Z-J , Peng Y-X , Wei L , Liu Y , Hu Y-H , Peng P , Wang J-M , Liu J-Y , Chen Z , Li G , Zheng Z-J , Qiu S-Q , Luo J , Ye C-J , Zhu S-Y , Zhong N-S , China Medical Treatment Expert Group for Covid-19 . 2020. Clinical characteristics of coronavirus disease 2019 in China. N Engl J Med 382:1708–1720. doi:10.1056/NEJMoa2002032 32109013 PMC7092819

[2] Wei X , Huang Z , Jiang L , Li Y , Zhang X , Leng Y , Jiang C . 2022. Charting the landscape of the environmental Exposome. iMeta 1:e50. doi:10.1002/imt2.50 PMC10989948 38867899

[3] Dong E , Du H , Gardner L . 2020. An interactive web-based dashboard to track COVID-19 in real time. Lancet Infect Dis 20:533–534. doi:10.1016/S1473-3099(20)30120-1 32087114 PMC7159018

[4] da Silva SJR , Alves da Silva CT , Mendes RPG , Pena L . 2020. Role of nonstructural proteins in the pathogenesis of SARS-CoV-2. J Med Virol 92:1427–1429. doi:10.1002/jmv.25858 32270884 PMC7262198

[5] Lu R , Zhao X , Li J , Niu P , Yang B , Wu H , Wang W , Song H , Huang B , Zhu N , Bi Y , Ma X , Zhan F , Wang L , Hu T , Zhou H , Hu Z , Zhou W , Zhao L , Chen J , Meng Y , Wang J , Lin Y , Yuan J , Xie Z , Ma J , Liu WJ , Wang D , Xu W , Holmes EC , Gao GF , Wu G , Chen W , Shi W , Tan W . 2020. Genomic characterisation and epidemiology of 2019 novel coronavirus: implications for virus origins and receptor binding. Lancet 395:565–574. doi:10.1016/S0140-6736(20)30251-8 32007145 PMC7159086

[6] Zhang Y-Z , Holmes EC . 2020. A genomic perspective on the origin and emergence of SARS-CoV-2. Cell 181:223–227. doi:10.1016/j.cell.2020.03.035 32220310 PMC7194821

[7] V’kovski P , Kratzel A , Steiner S , Stalder H , Thiel V . 2021. Coronavirus biology and replication: implications for SARS-CoV-2. Nat Rev Microbiol 19:155–170. doi:10.1038/s41579-020-00468-6 33116300 PMC7592455

[8] Sawicki SG , Sawicki DL . 1995. Coronaviruses use discontinuous extension for synthesis of subgenome-length negative strands. Adv Exp Med Biol 380:499–506. doi:10.1007/978-1-4615-1899-0_79 8830530

[9] Sola I , Almazán F , Zúñiga S , Enjuanes L . 2015. Continuous and discontinuous RNA synthesis in coronaviruses. Annu Rev Virol 2:265–288. doi:10.1146/annurev-virology-100114-055218 26958916 PMC6025776

[10] Lai MM , Stohlman SA . 1981. Comparative analysis of RNA genomes of mouse hepatitis viruses. J Virol 38:661–670. doi:10.1128/jvi.38.2.661-670.1981 6165837 PMC171196

[11] Schmidt N , Lareau CA , Keshishian H , Ganskih S , Schneider C , Hennig T , Melanson R , Werner S , Wei Y , Zimmer M , Ade J , Kirschner L , Zielinski S , Dölken L , Lander ES , Caliskan N , Fischer U , Vogel J , Carr SA , Bodem J , Munschauer M . 2021. The SARS-CoV-2 RNA-protein interactome in infected human cells. Nat Microbiol 6:339–353. doi:10.1038/s41564-020-00846-z 33349665 PMC7906908

[12] Flynn RA , Belk JA , Qi Y , Yasumoto Y , Wei J , Alfajaro MM , Shi Q , Mumbach MR , Limaye A , DeWeirdt PC , Schmitz CO , Parker KR , Woo E , Chang HY , Horvath TL , Carette JE , Bertozzi CR , Wilen CB , Satpathy AT . 2021. Discovery and functional interrogation of SARS-CoV-2 RNA-host protein interactions. Cell 184:2394–2411. doi:10.1016/j.cell.2021.03.012 33743211 PMC7951565

[13] Kamel W , Noerenberg M , Cerikan B , Chen H , Järvelin AI , Kammoun M , Lee JY , Shuai N , Garcia-Moreno M , Andrejeva A , Deery MJ , Johnson N , Neufeldt CJ , Cortese M , Knight ML , Lilley KS , Martinez J , Davis I , Bartenschlager R , Mohammed S , Castello A . 2021. Global analysis of protein-RNA interactions in SARS-CoV-2-infected cells reveals key regulators of infection. Mol Cell 81:2851–2867. doi:10.1016/j.molcel.2021.05.023 34118193 PMC8142890

[14] Lee S , Lee Y-S , Choi Y , Son A , Park Y , Lee K-M , Kim J , Kim J-S , Kim VN . 2021. The SARS-CoV-2 RNA interactome. Mol Cell 81:2838–2850. doi:10.1016/j.molcel.2021.04.022 33989516 PMC8075806

[15] Labeau A , Fery-Simonian L , Lefevre-Utile A , Pourcelot M , Bonnet-Madin L , Soumelis V , Lotteau V , Vidalain P-O , Amara A , Meertens L . 2022. Characterization and functional interrogation of the SARS-Cov-2 RNA Interactome. Cell Rep 39:110744. doi:10.1016/j.celrep.2022.110744 35477000 PMC9040432

[16] Pikovskaya O , Serganov AA , Polonskaia A , Serganov A , Patel DJ . 2009. Preparation and crystallization of Riboswitch-ligand complexes. Methods Mol Biol 540:115–128. doi:10.1007/978-1-59745-558-9_9 19381556

[17] Willforss J , Chawade A , Levander F . 2019. NormalyzerDE: online tool for improved normalization of omics expression data and high-sensitivity differential expression analysis. J Proteome Res 18:732–740. doi:10.1021/acs.jproteome.8b00523 30277078

[18] Leek JT , Johnson WE , Parker HS , Jaffe AE , Storey JD . 2012. The SVA package for removing batch effects and other unwanted variation in high-throughput experiments. Bioinformatics 28:882–883. doi:10.1093/bioinformatics/bts034 22257669 PMC3307112

[19] Dray S , Dufour A-B . 2007. The Ade4 package: Implementing the Duality diagram for ecologists. J. Stat. Soft 22:1–20. doi:10.18637/jss.v022.i04

[20] Nakamura T , Yamada KD , Tomii K , Katoh K . 2018. Parallelization of MAFFT for large-scale multiple sequence alignments. Bioinformatics 34:2490–2492. doi:10.1093/bioinformatics/bty121 29506019 PMC6041967

[21] Jiang C , Wang X , Li X , Inlora J , Wang T , Liu Q , Snyder M . 2018. Dynamic human environmental exposome revealed by longitudinal personal monitoring. Cell 175:277–291. doi:10.1016/j.cell.2018.08.060 30241608 PMC6472932

[22] Jassal B , Matthews L , Viteri G , Gong C , Lorente P , Fabregat A , Sidiropoulos K , Cook J , Gillespie M , Haw R , Loney F , May B , Milacic M , Rothfels K , Sevilla C , Shamovsky V , Shorser S , Varusai T , Weiser J , Wu G , Stein L , Hermjakob H , D’Eustachio P . 2020. The Reactome pathway Knowledgebase. Nucleic Acids Res 48:D498–D503. doi:10.1093/nar/gkz1031 31691815 PMC7145712

[23] Yu G , Wang L-G , Han Y , He Q-Y . 2012. clusterProfiler: An R package for comparing biological themes among Gene clusters. OMICS 16:284–287. doi:10.1089/omi.2011.0118 22455463 PMC3339379

[24] Liberzon A , Birger C , Thorvaldsdóttir H , Ghandi M , Mesirov JP , Tamayo P . 2015. The molecular signatures database (Msigdb) hallmark Gene set collection. Cell Syst 1:417–425. doi:10.1016/j.cels.2015.12.004 26771021 PMC4707969

[25] Szklarczyk D , Gable AL , Lyon D , Junge A , Wyder S , Huerta-Cepas J , Simonovic M , Doncheva NT , Morris JH , Bork P , Jensen LJ , Mering C von . 2019. STRING V11: Protein-protein Association networks with increased coverage, supporting functional discovery in genome-wide experimental Datasets. Nucleic Acids Res 47:D607–D613. doi:10.1093/nar/gky1131 30476243 PMC6323986

[26] Caudron-Herger M , Jansen RE , Wassmer E , Diederichs S . 2021. RBP2GO: a comprehensive pan-species database on RNA-binding proteins, their interactions and functions. Nucleic Acids Res 49:D425–D436. doi:10.1093/nar/gkaa1040 33196814 PMC7778890

[27] Pan X , Fang Y , Li X , Yang Y , Shen HB . 2020. Rbpsuite: RNA-protein binding sites prediction suite based on deep learning. BMC Genomics 21:884. doi:10.1186/s12864-020-07291-6 33297946 PMC7724624

[28] Pan X , Rijnbeek P , Yan J , Shen H-B . 2018. Prediction of RNA-protein sequence and structure binding preferences using deep Convolutional and recurrent neural networks. BMC Genomics 19:511. doi:10.1186/s12864-018-4889-1 29970003 PMC6029131

[29] Shannon P , Markiel A , Ozier O , Baliga NS , Wang JT , Ramage D , Amin N , Schwikowski B , Ideker T . 2003. Cytoscape: A software environment for integrated models of Biomolecular interaction networks. Genome Res 13:2498–2504. doi:10.1101/gr.1239303 14597658 PMC403769

[30] Wickham H . 2011. ggplot2. WIREs Comp Stat 3:180–185. doi:10.1002/wics.147

[31] Kolde R , Kolde MR . 2015. Package 'Pheatmap' R package 1:790.

[32] Gu Z , Eils R , Schlesner M . 2016. Complex Heatmaps reveal patterns and correlations in multidimensional Genomic data. Bioinformatics 32:2847–2849. doi:10.1093/bioinformatics/btw313 27207943

[33] Conway JR , Lex A , Gehlenborg N . 2017. Upsetr: An R package for the visualization of intersecting sets and their properties. Bioinformatics 33:2938–2940. doi:10.1093/bioinformatics/btx364 28645171 PMC5870712

[34] Chan AP , Choi Y , Schork NJ . 2020. Conserved Genomic terminals of SARS-Cov-2 as Coevolving functional elements and potential therapeutic targets. mSphere 5:e00754-20. doi:10.1128/mSphere.00754-20 33239366 PMC7690956

[35] Park JH , Moon J . 2022. Conserved 3' UTR of severe acute respiratory syndrome Coronavirus 2: Potential therapeutic targets. Front Genet 13:893141. doi:10.3389/fgene.2022.893141 35846120 PMC9280349

[36] Thi Nhu Thao T , Labroussaa F , Ebert N , V’kovski P , Stalder H , Portmann J , Kelly J , Steiner S , Holwerda M , Kratzel A , Gultom M , Schmied K , Laloli L , Hüsser L , Wider M , Pfaender S , Hirt D , Cippà V , Crespo-Pomar S , Schröder S , Muth D , Niemeyer D , Corman VM , Müller MA , Drosten C , Dijkman R , Jores J , Thiel V . 2020. Rapid reconstruction of SARS-Cov-2 using a synthetic Genomics platform. Nature 582:561–565. doi:10.1038/s41586-020-2294-9 32365353

[37] Kim D , Lee JY , Yang JS , Kim JW , Kim VN , Chang H . 2020. The architecture of SARS-CoV-2 transcriptome. Cell 181:914–921. doi:10.1016/j.cell.2020.04.011 32330414 PMC7179501

[38] Wu F , Zhao S , Yu B , Chen YM , Wang W , Song ZG , Hu Y , Tao ZW , Tian JH , Pei YY , Yuan ML , Zhang YL , Dai FH , Liu Y , Wang QM , Zheng JJ , Xu L , Holmes EC , Zhang YZ . 2020. A new coronavirus associated with human respiratory disease in China. Nature 579:265–269. doi:10.1038/s41586-020-2008-3 32015508 PMC7094943

[39] Pancer K , Milewska A , Owczarek K , Dabrowska A , Kowalski M , Łabaj PP , Branicki W , Sanak M , Pyrc K . 2020. The SARS-Cov-2 Orf10 is not essential in vitro or in vivo in humans. PLoS Pathog 16:e1008959. doi:10.1371/journal.ppat.1008959 33301543 PMC7755277

[40] Yao H , Lu X , Chen Q , Xu K , Chen Y , Cheng M , Chen K , Cheng L , Weng T , Shi D , Liu F , Wu Z , Xie M , Wu H , Jin C , Zheng M , Wu N , Jiang C , Li L . 2020. Patient-Derived SARS-CoV-2 mutations impact viral replication dynamics and infectivity in vitro and with clinical implications in vivo. Cell Discov 6:76. doi:10.1038/s41421-020-00226-1 33298872 PMC7595057

[41] Baserga S , Steitz J 1993. The RNA world. : Cold Spring Harbor Laboratory Press.

[42] Gélinas J-F , Clerzius G , Shaw E , Gatignol A . 2011. Enhancement of replication of RNA viruses by ADAR1 via RNA editing and inhibition of RNA-activated protein kinase. J Virol 85:8460–8466. doi:10.1128/JVI.00240-11 21490091 PMC3165853

[43] Di Giorgio S , Martignano F , Torcia MG , Mattiuz G , Conticello SG . 2020. Evidence for host-dependent RNA editing in the Transcriptome of SARS-Cov-2. Sci Adv 6:eabb5813. doi:10.1126/sciadv.abb5813 32596474 PMC7299625

[44] Anderson EC , Hunt SL , Jackson RJ . 2007. Internal initiation of translation from the human rhinovirus-2 internal ribosome entry site requires the binding of Unr to two distinct sites on the 5’ untranslated region. J Gen Virol 88:3043–3052. doi:10.1099/vir.0.82463-0 17947529

[45] Boussadia O , Niepmann M , Créancier L , Prats A-C , Dautry F , Jacquemin-Sablon H . 2003. Unr is required in vivo for efficient initiation of translation from the internal ribosome entry sites of both rhinovirus and poliovirus. J Virol 77:3353–3359. doi:10.1128/jvi.77.6.3353-3359.2003 12610110 PMC149491

[46] Xiong G , Stewart RL , Chen J , Gao T , Scott TL , Samayoa LM , O’Connor K , Lane AN , Xu R . 2018. Collagen Prolyl 4-hydroxylase 1 is essential for HIF-1Α Stabilization and TNBC Chemoresistance. Nat Commun 9:4456. doi:10.1038/s41467-018-06893-9 30367042 PMC6203834

[47] Serebrovska ZO , Chong EY , Serebrovska TV , Tumanovska LV , Xi L . 2020. Hypoxia, HIF-1α, and COVID-19: from pathogenic factors to potential therapeutic targets. Acta Pharmacol Sin 41:1539–1546. doi:10.1038/s41401-020-00554-8 33110240 PMC7588589

[48] Aviner R , Li KH , Frydman J , Andino R . 2021. Cotranslational prolyl hydroxylation is essential for flavivirus biogenesis. Nature 596:558–564. doi:10.1038/s41586-021-03851-2 34408324 PMC8789550

[49] Zimmer MM , Kibe A , Rand U , Pekarek L , Ye L , Buck S , Smyth RP , Cicin-Sain L , Caliskan N . 2021. The short Isoform of the host antiviral protein ZAP acts as an inhibitor of SARS-Cov-2 programmed Ribosomal Frameshifting. Nat Commun 12:7193. doi:10.1038/s41467-021-27431-0 34893599 PMC8664833

[50] Nchioua R , Kmiec D , Müller JA , Conzelmann C , Groß R , Swanson CM , Neil SJD , Stenger S , Sauter D , Münch J , Sparrer KMJ , Kirchhoff F . 2020. SARS-Cov-2 is restricted by zinc finger antiviral protein despite Preadaptation to the low-Cpg environment in humans. mBio 11:e01930-20. doi:10.1128/mBio.01930-20 33067384 PMC7569149

[51] Hayakawa S , Shiratori S , Yamato H , Kameyama T , Kitatsuji C , Kashigi F , Goto S , Kameoka S , Fujikura D , Yamada T , Mizutani T , Kazumata M , Sato M , Tanaka J , Asaka M , Ohba Y , Miyazaki T , Imamura M , Takaoka A . 2011. ZAPS is a potent stimulator of signaling mediated by the RNA helicase RIG-I during antiviral responses. Nat Immunol 12:37–44. doi:10.1038/ni.1963 21102435

[52] Cuevas RA , Ghosh A , Wallerath C , Hornung V , Coyne CB , Sarkar SN . 2016. MOV10 provides antiviral activity against RNA viruses by enhancing RIG-I-MAVS-independent IFN induction. J Immunol 196:3877–3886. doi:10.4049/jimmunol.1501359 27016603 PMC4868630

[53] Wang L , Sola I , Enjuanes L , Zuñiga S . 2021. Mov10 Helicase interacts with Coronavirus Nucleocapsid protein and has antiviral activity. mBio 12:e0131621. doi:10.1128/mBio.01316-21 34517762 PMC8546642

[54] Dauber B , Wolff T . 2009. Activation of the antiviral kinase PKR and viral countermeasures. Viruses 1:523–544. doi:10.3390/v1030523 21994559 PMC3185532

[55] Liu Y , Wang M , Cheng A , Yang Q , Wu Y , Jia R , Liu M , Zhu D , Chen S , Zhang S , Zhao X-X , Huang J , Mao S , Ou X , Gao Q , Wang Y , Xu Z , Chen Z , Zhu L , Luo Q , Liu Y , Yu Y , Zhang L , Tian B , Pan L , Rehman MU , Chen X . 2020. The role of host eIF2α in viral infection. Virol J 17:112. doi:10.1186/s12985-020-01362-6 32703221 PMC7376328

[56] Wang Y , Fan Y , Huang Y , Du T , Liu Z , Huang D , Wang Y , Wang N , Zhang P . 2021. Trim28 regulates SARS-Cov-2 cell entry by targeting Ace2. Cell Signal 85:110064. doi:10.1016/j.cellsig.2021.110064 34146659 PMC8213541

[57] Gao B , Gong X , Fang S , Weng W , Wang H , Chu H , Sun Y , Meng C , Tan L , Song C , Qiu X , Liu W , Forlenza M , Ding C , Liao Y . 2021. Inhibition of anti-viral stress granule formation by Coronavirus Endoribonuclease Nsp15 ensures efficient virus replication. PLoS Pathog 17:e1008690. doi:10.1371/journal.ppat.1008690 33635931 PMC7946191

[58] Lu S , Ye Q , Singh D , Cao Y , Diedrich JK , Yates JR , Villa E , Cleveland DW , Corbett KD . 2021. The SARS-CoV-2 nucleocapsid phosphoprotein forms mutually exclusive condensates with RNA and the membrane-associated M protein. Nat Commun 12: 502. doi:10.1038/s41467-020-20768-y 33479198 PMC7820290

[59] Luo L , Li Z , Zhao T , Ju X , Ma P , Jin B , Zhou Y , He S , Huang J , Xu X , Zou Y , Li P , Liang A , Liu J , Chi T , Huang X , Ding Q , Jin Z , Huang C , Zhang Y . 2021. SARS-CoV-2 nucleocapsid protein phase separates with G3BPs to disassemble stress granules and facilitate viral production. Sci Bull (Beijing) 66:1194–1204. doi:10.1016/j.scib.2021.01.013 33495715 PMC7816596

[60] Yang P , Mathieu C , Kolaitis R-M , Zhang P , Messing J , Yurtsever U , Yang Z , Wu J , Li Y , Pan Q , Yu J , Martin EW , Mittag T , Kim HJ , Taylor JP . 2020. G3Bp1 is a tunable switch that triggers phase separation to assemble stress granules. Cell 181:325–345. doi:10.1016/j.cell.2020.03.046 32302571 PMC7448383

[61] Zheng Z-Q , Wang S-Y , Xu Z-S , Fu Y-Z , Wang Y-Y . 2021. SARS-CoV-2 nucleocapsid protein impairs stress granule formation to promote viral replication. Cell Discov 7:38. doi:10.1038/s41421-021-00275-0 34035218 PMC8147577

[62] Nabeel-Shah S , Lee H , Ahmed N , Burke GL , Farhangmehr S , Ashraf K , Pu S , Braunschweig U , Zhong G , Wei H , Tang H , Yang J , Marcon E , Blencowe BJ , Zhang Z , Greenblatt JF . 2022. SARS-Cov-2 Nucleocapsid protein binds host mRNAs and attenuates stress granules to impair host stress response. iScience 25:103562. doi:10.1016/j.isci.2021.103562 34901782 PMC8642831

[63] Dreyfuss G , Matunis MJ , Piñol-Roma S , Burd CG . 1993. HnRNP proteins and the biogenesis of mRNA. Annu Rev Biochem 62:289–321. doi:10.1146/annurev.bi.62.070193.001445 8352591

[64] Zhou F , Wan Q , Chen S , Chen Y , Wang P-H , Yao X , He M-L . 2021. Attenuating innate immunity and facilitating β-coronavirus infection by nsp1 of SARS-CoV-2 through specific redistributing hnRNP A2/B1 cellular localization. Signal Transduct Target Ther 6:371. doi:10.1038/s41392-021-00786-y 34702797 PMC8546379

[65] Kumar R , Khandelwal N , Chander Y , Nagori H , Verma A , Barua A , Godara B , Pal Y , Gulati BR , Tripathi BN , Barua S , Kumar N . 2022. S-Adenosylmethionine-dependent Methyltransferase inhibitor Dznep blocks transcription and translation of SARS-Cov-2 genome with a low tendency to select for drug-resistant viral variants. Antiviral Res 197:105232. doi:10.1016/j.antiviral.2021.105232 34968527 PMC8714615

[66] Frick DN , Lam AMI . 2006. Understanding helicases as a means of virus control. Curr Pharm Des 12:1315–1338. doi:10.2174/138161206776361147 16611118 PMC3571686

[67] Brai A , Martelli F , Riva V , Garbelli A , Fazi R , Zamperini C , Pollutri A , Falsitta L , Ronzini S , Maccari L , Maga G , Giannecchini S , Botta M . 2019. DDX3X helicase inhibitors as a new strategy to fight the West Nile virus infection. J Med Chem 62:2333–2347. doi:10.1021/acs.jmedchem.8b01403 30721061

[68] Xu L , Khadijah S , Fang S , Wang L , Tay FPL , Liu DX . 2010. The cellular RNA helicase DDX1 interacts with coronavirus nonstructural protein 14 and enhances viral replication. J Virol 84:8571–8583. doi:10.1128/JVI.00392-10 20573827 PMC2918985

[69] Cheng W , Chen G , Jia H , He X , Jing Z . 2018. Ddx5 RNA Helicases: Emerging roles in viral infection. Int J Mol Sci 19:1122. doi:10.3390/ijms19041122 29642538 PMC5979547

[70] Ariumi Y . 2022. Host cellular RNA Helicases regulate SARS-Cov-2 infection. J Virol 96:e0000222. doi:10.1128/jvi.00002-22 35107372 PMC8941876

[71] Ciccosanti F , Di Rienzo M , Romagnoli A , Colavita F , Refolo G , Castilletti C , Agrati C , Brai A , Manetti F , Botta L , Capobianchi MR , Ippolito G , Piacentini M , Fimia GM . 2021. Proteomic analysis identifies the RNA Helicase Ddx3X as a host target against SARS-Cov-2 infection. Antiviral Res 190:105064. doi:10.1016/j.antiviral.2021.105064 33781803 PMC7997689

[72] Choksupmanee O , Tangkijthavorn W , Hodge K , Trisakulwattana K , Phornsiricharoenphant W , Narkthong V , Tulakarnwong S , Ngamphiw C , Tongsima S , Chimnaronk S . 2021. Specific interaction of Ddx6 with an RNA hairpin in the 3’ UTR of the Dengue virus genome mediates G1 phase arrest. J Virol 95:e0051021. doi:10.1128/JVI.00510-21 34132569 PMC8354244

[73] Ficarelli M , Neil SJD , Swanson CM . 2021. Targeted restriction of viral gene expression and replication by the ZAP antiviral system. Annu Rev Virol 8:265–283. doi:10.1146/annurev-virology-091919-104213 34129371

[74] Watanabe Y , Ohtaki N , Hayashi Y , Ikuta K , Tomonaga K , Basler CF . 2009. Autogenous translational regulation of the Borna disease virus negative control factor X from polycistronic mRNA using host RNA helicases. PLoS Pathog 5:e1000654. doi:10.1371/journal.ppat.1000654 19893625 PMC2766071

[75] Langfelder P , Horvath S . 2008. WGCNA: An R package for weighted correlation network analysis. BMC Bioinformatics 9:559. doi:10.1186/1471-2105-9-559 19114008 PMC2631488

[76] Bitko V , Musiyenko A , Bayfield MA , Maraia RJ , Barik S . 2008. Cellular La protein shields nonsegmented negative-strand RNA viral leader RNA from RIG-I and enhances virus growth by diverse mechanisms. J Virol 82:7977–7987. doi:10.1128/JVI.02762-07 18550659 PMC2519562

[77] Kash JC , Cunningham DM , Smit MW , Park Y , Fritz D , Wilusz J , Katze MG . 2002. Selective translation of eukaryotic mRNAs: functional molecular analysis of GRSF-1, a positive regulator of influenza virus protein synthesis. J Virol 76:10417–10426. doi:10.1128/jvi.76.20.10417-10426.2002 12239318 PMC136571

[78] Zolfaghari Emameh R , Nosrati H , Eftekhari M , Falak R , Khoshmirsafa M . 2020. Expansion of single cell transcriptomics data of SARS-CoV infection in human bronchial epithelial cells to COVID-19. Biol Proced Online 22:16. doi:10.1186/s12575-020-00127-3 32754004 PMC7377208

[79] Cai T , Yu Z , Wang Z , Liang C , Richard S . 2021. Arginine methylation of SARS-Cov-2 Nucleocapsid protein regulates RNA binding, its ability to suppress stress granule formation, and viral replication. J Biol Chem 297:100821. doi:10.1016/j.jbc.2021.100821 34029587 PMC8141346

[80] Wang J-Y , Lu A-Q . 2021. The biological function of m6A reader YTHDF2 and its role in human disease. Cancer Cell Int 21:109. doi:10.1186/s12935-021-01807-0 33593354 PMC7885220

[81] Liu J , Xu Y-P , Li K , Ye Q , Zhou H-Y , Sun H , Li X , Yu L , Deng Y-Q , Li R-T , Cheng M-L , He B , Zhou J , Li X-F , Wu A , Yi C , Qin C-F . 2021. The M6a methylome of SARS-CoV-2 in host cells. Cell Res 31:404–414. doi:10.1038/s41422-020-00465-7 33510385 PMC8115241

[82] Shi D , Shi H , Sun D , Chen J , Zhang X , Wang X , Zhang J , Ji Z , Liu J , Cao L , Zhu X , Yuan J , Dong H , Wang X , Chang T , Liu Y , Feng L . 2017. Nucleocapsid interacts with Npm1 and protects it from proteolytic cleavage, enhancing cell survival, and is involved in PEDV growth. Sci Rep 7:39700. doi:10.1038/srep39700 28045037 PMC5206633

[83] Zhou B , Wu F , Han J , Qi F , Ni T , Qian F . 2019. Exploitation of nuclear protein SFPQ by the encephalomyocarditis virus to facilitate its replication. Biochem Biophys Res Commun 510:65–71. doi:10.1016/j.bbrc.2019.01.032 30661786

[84] Landeras-Bueno S , Jorba N , Pérez-Cidoncha M , Ortín J . 2011. The splicing factor Proline-Glutamine rich (SFPQ/PSF) is involved in influenza virus transcription. PLoS Pathog 7:e1002397. doi:10.1371/journal.ppat.1002397 22114566 PMC3219729

[85] Lee N , Yario TA , Gao JS , Steitz JA . 2016. EBV noncoding RNA EBER2 interacts with host RNA-binding proteins to regulate viral gene expression. Proc Natl Acad Sci U S A 113:3221–3226. doi:10.1073/pnas.1601773113 26951683 PMC4812724

[86] Yuan S , Peng L , Park JJ , Hu Y , Devarkar SC , Dong MB , Shen Q , Wu S , Chen S , Lomakin IB , Xiong Y . 2020. Nonstructural protein 1 of SARS-CoV-2 is a potent pathogenicity factor Redirecting host protein synthesis machinery toward viral RNA. Mol Cell 80:1055–1066. doi:10.1016/j.molcel.2020.10.034 33188728 PMC7833686

[87] Tiku V , Tan M-W , Dikic I . 2020. Mitochondrial functions in infection and immunity. Trends Cell Biol 30:263–275. doi:10.1016/j.tcb.2020.01.006 32200805 PMC7126537

[88] Ganji R , Reddy PH . 2020. Impact of COVID-19 on mitochondrial-based immunity in aging and age-related diseases. Front Aging Neurosci 12:614650. doi:10.3389/fnagi.2020.614650 33510633 PMC7835331

[89] Gatti P , Ilamathi HS , Todkar K , Germain M . 2020. Mitochondria targeted viral replication and survival strategies-prospective on SARS-Cov-2. Front Pharmacol 11:578599. doi:10.3389/fphar.2020.578599 32982760 PMC7485471

[90] Gordon DE , Jang GM , Bouhaddou M , Xu J , Obernier K , White KM , O’Meara MJ , Rezelj VV , Guo JZ , Swaney DL , Tummino TA , Hüttenhain R , Kaake RM , Richards AL , Tutuncuoglu B , Foussard H , Batra J , Haas K , Modak M , Kim M , Haas P , Polacco BJ , Braberg H , Fabius JM , Eckhardt M , Soucheray M , Bennett MJ , Cakir M , McGregor MJ , Li Q , Meyer B , Roesch F , Vallet T , Mac Kain A , Miorin L , Moreno E , Naing ZZC , Zhou Y , Peng S , Shi Y , Zhang Z , Shen W , Kirby IT , Melnyk JE , Chorba JS , Lou K , Dai SA , Barrio-Hernandez I , Memon D , Hernandez-Armenta C , Lyu J , Mathy CJP , Perica T , Pilla KB , Ganesan SJ , Saltzberg DJ , Rakesh R , Liu X , Rosenthal SB , Calviello L , Venkataramanan S , Liboy-Lugo J , Lin Y , Huang X-P , Liu Y , Wankowicz SA , Bohn M , Safari M , Ugur FS , Koh C , Savar NS , Tran QD , Shengjuler D , Fletcher SJ , O’Neal MC , Cai Y , Chang JCJ , Broadhurst DJ , Klippsten S , Sharp PP , Wenzell NA , Kuzuoglu-Ozturk D , Wang H-Y , Trenker R , Young JM , Cavero DA , Hiatt J , Roth TL , Rathore U , Subramanian A , Noack J , Hubert M , Stroud RM , Frankel AD , Rosenberg OS , Verba KA , Agard DA , Ott M , Emerman M , Jura N , von Zastrow M , Verdin E , Ashworth A , Schwartz O , d’Enfert C , Mukherjee S , Jacobson M , Malik HS , Fujimori DG , Ideker T , Craik CS , Floor SN , Fraser JS , Gross JD , Sali A , Roth BL , Ruggero D , Taunton J , Kortemme T , Beltrao P , Vignuzzi M , García-Sastre A , Shokat KM , Shoichet BK , Krogan NJ . 2020. A SARS-CoV-2 protein interaction map reveals targets for drug repurposing. Nature 583:459–468. doi:10.1038/s41586-020-2286-9 32353859 PMC7431030

[91] Banerjee AK , Blanco MR , Bruce EA , Honson DD , Chen LM , Chow A , Bhat P , Ollikainen N , Quinodoz SA , Loney C , Thai J , Miller ZD , Lin AE , Schmidt MM , Stewart DG , Goldfarb D , De Lorenzo G , Rihn SJ , Voorhees RM , Botten JW , Majumdar D , Guttman M . 2020. SARS-CoV-2 disrupts splicing, translation, and protein trafficking to suppress host defenses. Cell 183:1325–1339. doi:10.1016/j.cell.2020.10.004 33080218 PMC7543886

[92] Savastano A , Ibáñez de Opakua A , Rankovic M , Zweckstetter M . 2020. Nucleocapsid protein of SARS-Cov-2 phase separates into RNA-rich polymerase-containing condensates. Nat Commun 11:6041. doi:10.1038/s41467-020-19843-1 33247108 PMC7699647

[93] Jungreis I , Sealfon R , Kellis M . 2021. SARS-Cov-2 Gene content and COVID-19 Mutation impact by comparing 44 Sarbecovirus Genomes. Nat Commun 12:2642. doi:10.1038/s41467-021-22905-7 33976134 PMC8113528

[94] Chang X , Ismail NI , Rahman A , Xu D , Chan RWY , Ong SG , Ong SB . 2023. Long COVID-19 and the heart: is cardiac mitochondria the missing link? Antioxid Redox Signal 38:599–618. doi:10.1089/ars.2022.0126 36053670 PMC10025846

[95] Hackbart M , Deng X , Baker SC . 2020. Coronavirus endoribonuclease targets viral polyuridine sequences to evade activating host sensors. Proc Natl Acad Sci U S A 117:8094–8103. doi:10.1073/pnas.1921485117 32198201 PMC7149396

