# Supplementary material for: High-sensitivity profiling of SARS-CoV-2 noncoding region–host protein interactome reveals the potential regulatory role of negative-sense viral RNA
Source: mSystems. 2023 Jun 14;8(4):e00135-23. doi: 10.1128/msystems.00135-23 (PMC10469612; doi:10.1128/msystems.00135-23)

Figure S1

A

Variant	Sample[1]	Pango lineage	QC passed[2]
Alpha	3161	B.1.1.7	2350
Beta	805	B.1.351	718
Gamma	1073	P.1	976
Delta	2652	B.1.617.2	1196
Omicron	10949	B.1.1.529, BA.X	430

[1] The min length of the genome was set as 29890 bp, except for Beta variant was set as 29850 bp, because the max length of Beta variant was less than 29890 bp.
[2] Sequences with >1% of ambiguous bases were removed.

B

Label	Position	Average pi
ORF8	27894-28259	0.00749703
N	28274-29533	0.00530035
3' UTR	29675-29903	0.00460326
S	21563-25384	0.00354704
ORF7b	27756-27877	0.00339287
5' UTR	1-265	0.00321101
ORF7a	27394-27759	0.00315498
ORF3a	25393-26220	0.00273322
ORF6	27202-27387	0.00243431
E	26245-26472	0.00172148
M	26523-27191	0.00131433
ORF1ab	266-21555	0.00101929
ORF10	29588-29674	0.00046292

C

Position of TRS-L/B (ACGAAC)	CDS region	Position	Number of bases in the interval
70-75	5′ UTR	1-265	/
21556-21561	S	21563-25384	1
25385-25390	ORF3a	25393-26220	2
26237-26242	E	26245-26472	2
26473-26478	M	26523-27191	44
27041-27046	ORF6	27202-27387	155
27388-27393	ORF7a	27394-27759	0
27888-27893	ORF8	27894-25259	0
28260-28265	N	28274-29533	8

D

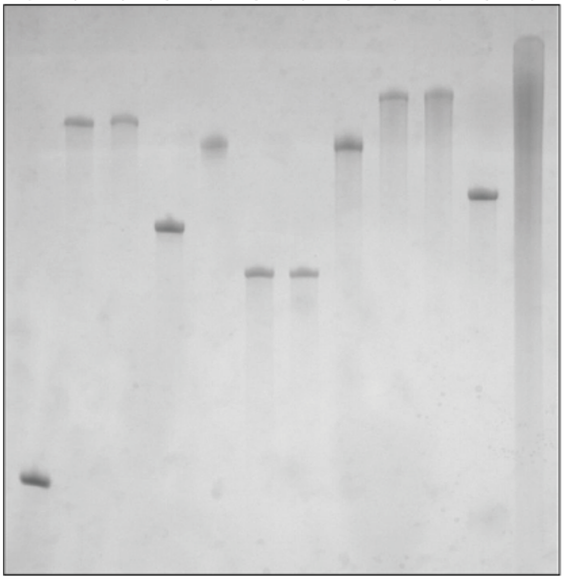

Supplement: Fig. S1 — Design and validation of NRCs. (A) SARS-CoV-2 sequence information, as downloaded from the NCBI Virus database. (B) Average nucleotide diversity (pi value) of different regions of SARS-CoV-2. (C) Position information of TRS-L/TRS-Bs in the SARS-CoV-2 genome. (D) Gel graphs of 12 NRCs after purification. [file msystems.00135-23-s0001.pdf]
