# Supplementary figures and images for: High-sensitivity profiling of SARS-CoV-2 noncoding region–host protein interactome reveals the potential regulatory role of negative-sense viral RNA
Source: mSystems. 2023 Jun 14;8(4):e00135-23. doi: 10.1128/msystems.00135-23 (PMC10469612; doi:10.1128/msystems.00135-23)

Figure S2

Calu-3 cells

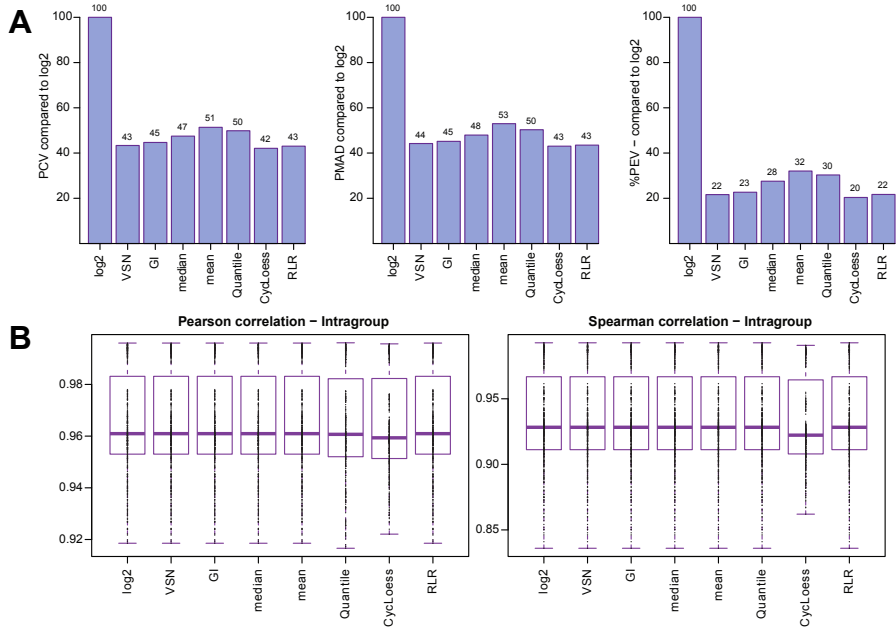

Huh7 cells

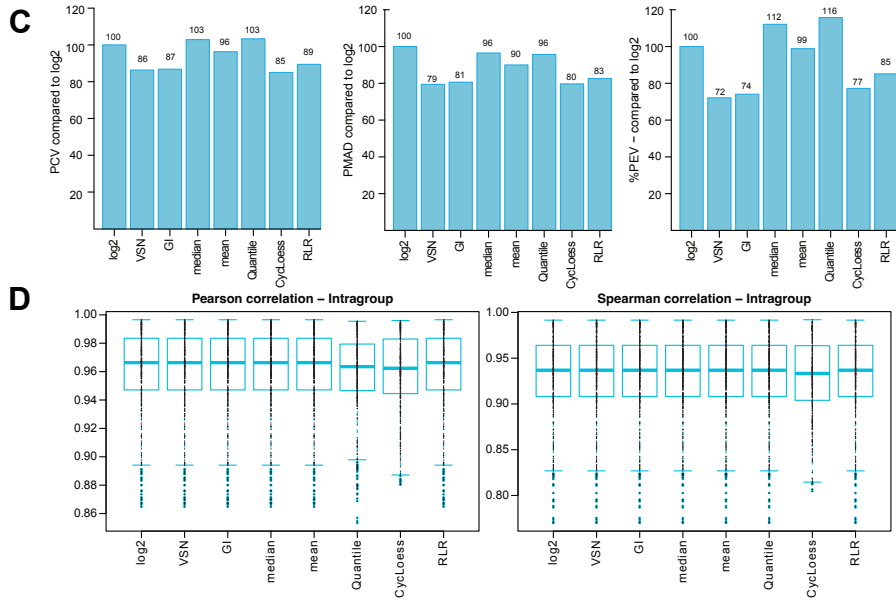

HEK293T cells

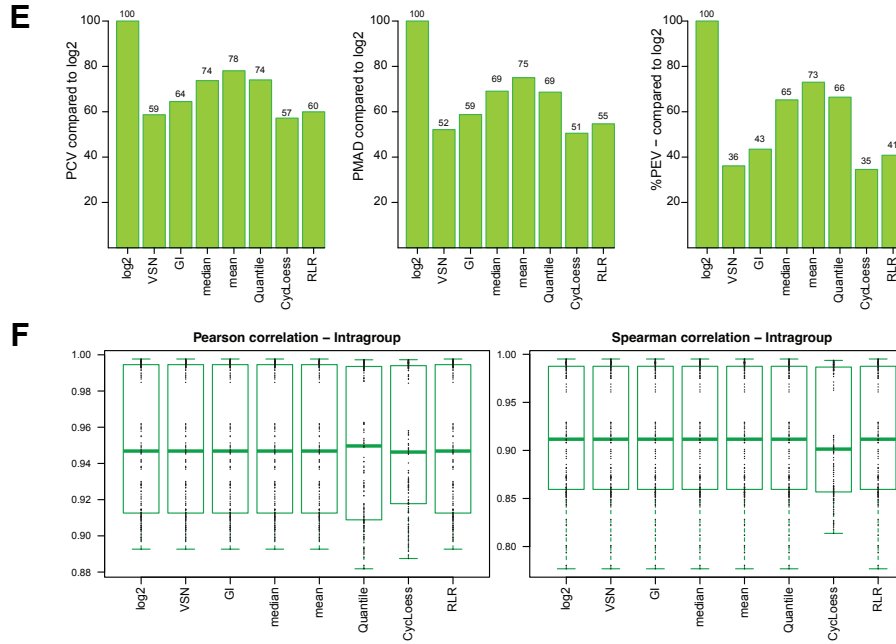

Supplement: Fig. S2 — Systematic evaluation of different normalization methods. (A) Bar plots demonstrating the PCV (left), PMAD (middle), and PEV (right) statistics of different normalization methods when compared with the log2 transformation in Calu-3 cells (the lower the better). (B) Box plots showing the Pearson correlation (left) and Spearman correlation (right) between raw data and normalized data in Calu-3 cells. (C) Bar plots demonstrating the PCV (left), PMAD (middle), and PEV (right) statistics of different normalization methods compared with the standard log2 transformation in Huh7 cells. (D) Box plots showing the Pearson correlation (left) and Spearman correlation (right) between raw data and normalized data in Huh7 cells. (E) Bar plots demonstrating the PCV (left), PMAD (middle), and PEV (right) statistics of different normalization methods when compared with the log2 transformation in HEK293T cells (The lower the better). (F) Box plots showing the Pearson correlation (left) and Spearman correlation (right) between raw data and normalized data in HEK293T cells. [file msystems.00135-23-s0002.pdf]

Figure S3

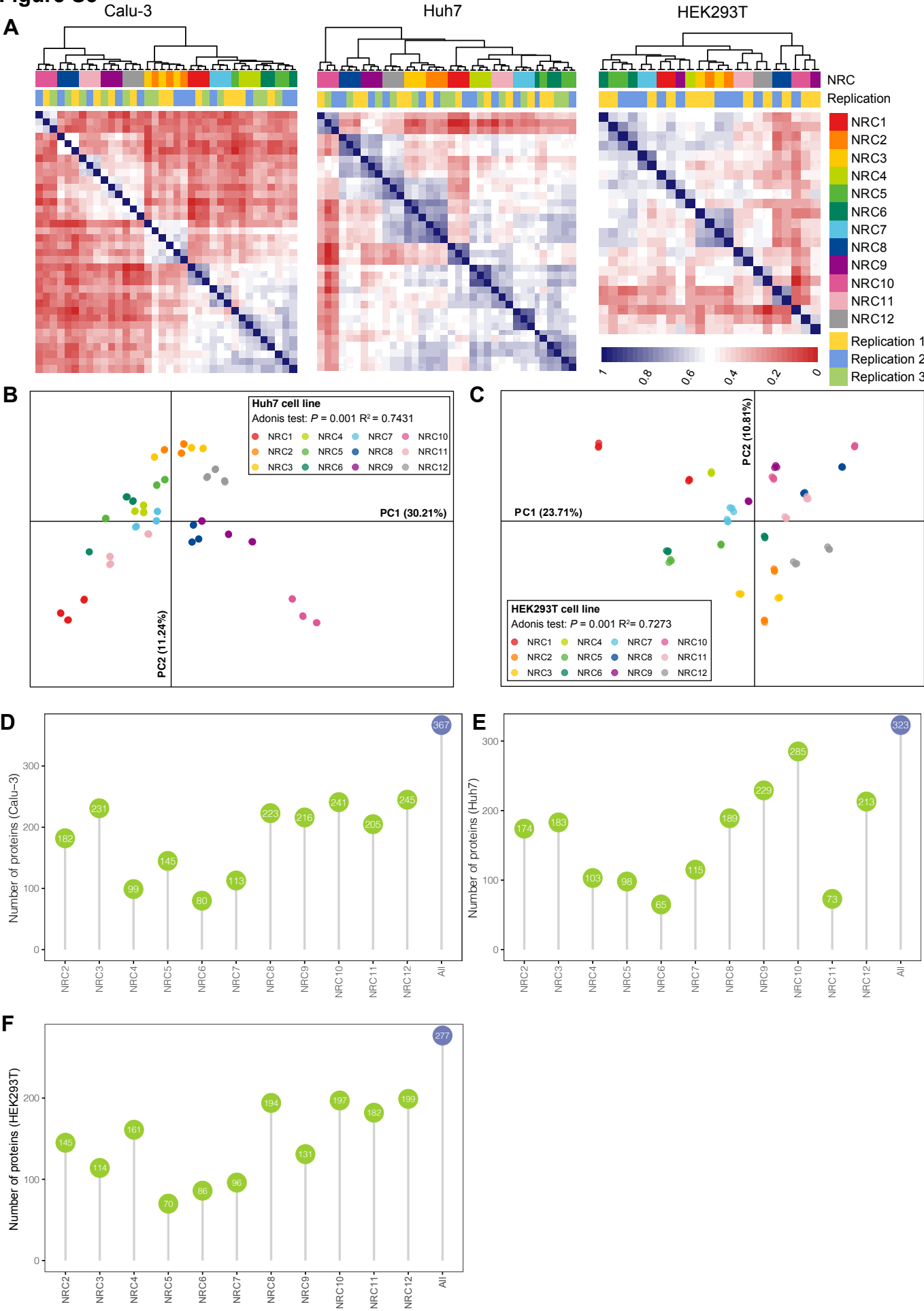

Supplement: Fig. S3 — Overview of the ncrRNA interactomes in cell lines. (A) Heatmaps of the interactomes revealing that the biological and technical replicates of NRCs are highly correlated in Calu-3 (left), Huh7 (middle), and HEK293T (right) cells. (B, C) PCA analyses show ncrRNA interactomes’ patterns in Huh7 (B) and HEK293T (C). Dots represent different samples. Colors indicate different NRCs. The adonis test was used to determine statistical significance. (D–F) Overview of the numbers of host proteins bound to different NRCs in Calu-3 (D), Huh7 (E), and HEK293T (F) cells. See also Table S1. [file msystems.00135-23-s0003.pdf]

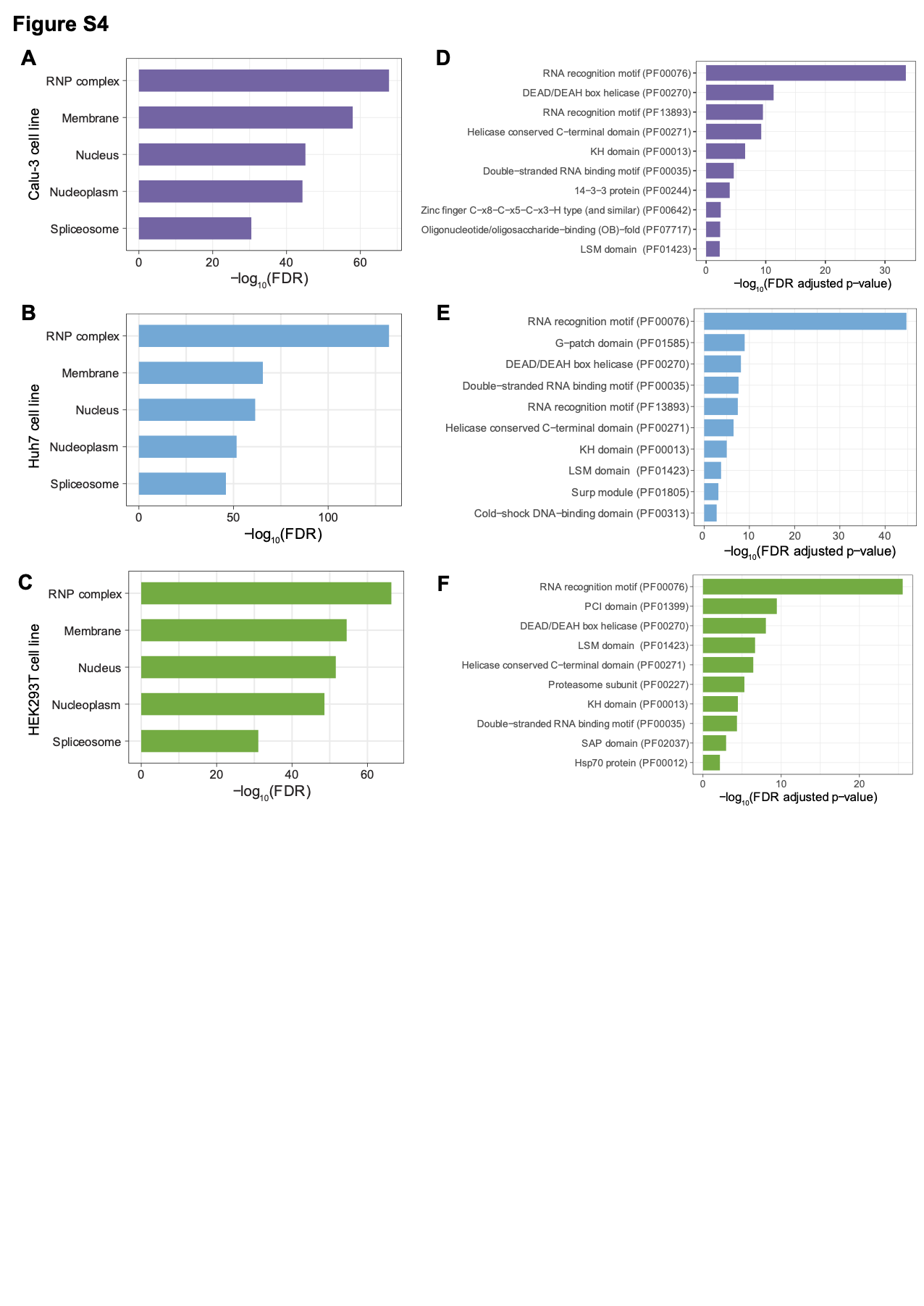

Supplement: Fig. S4 — Cell components and domain analysis of SARS-CoV-2 ncrRNA interactome. (A–C) Gene ontology cellular component analysis of host proteins enriched in SARS-CoV-2 ncrRNA interactomes in Calu-3 (A), Huh7 (B), and HEK293T (C) cells. (D–F) Protein domain enrichment analyses of host proteins enriched in SARS-CoV-2 ncrRNA interactomes in Calu-3 (D), Huh7 (E), and HEK293T (F) cells. [file msystems.00135-23-s0004.tif]

Figure S5

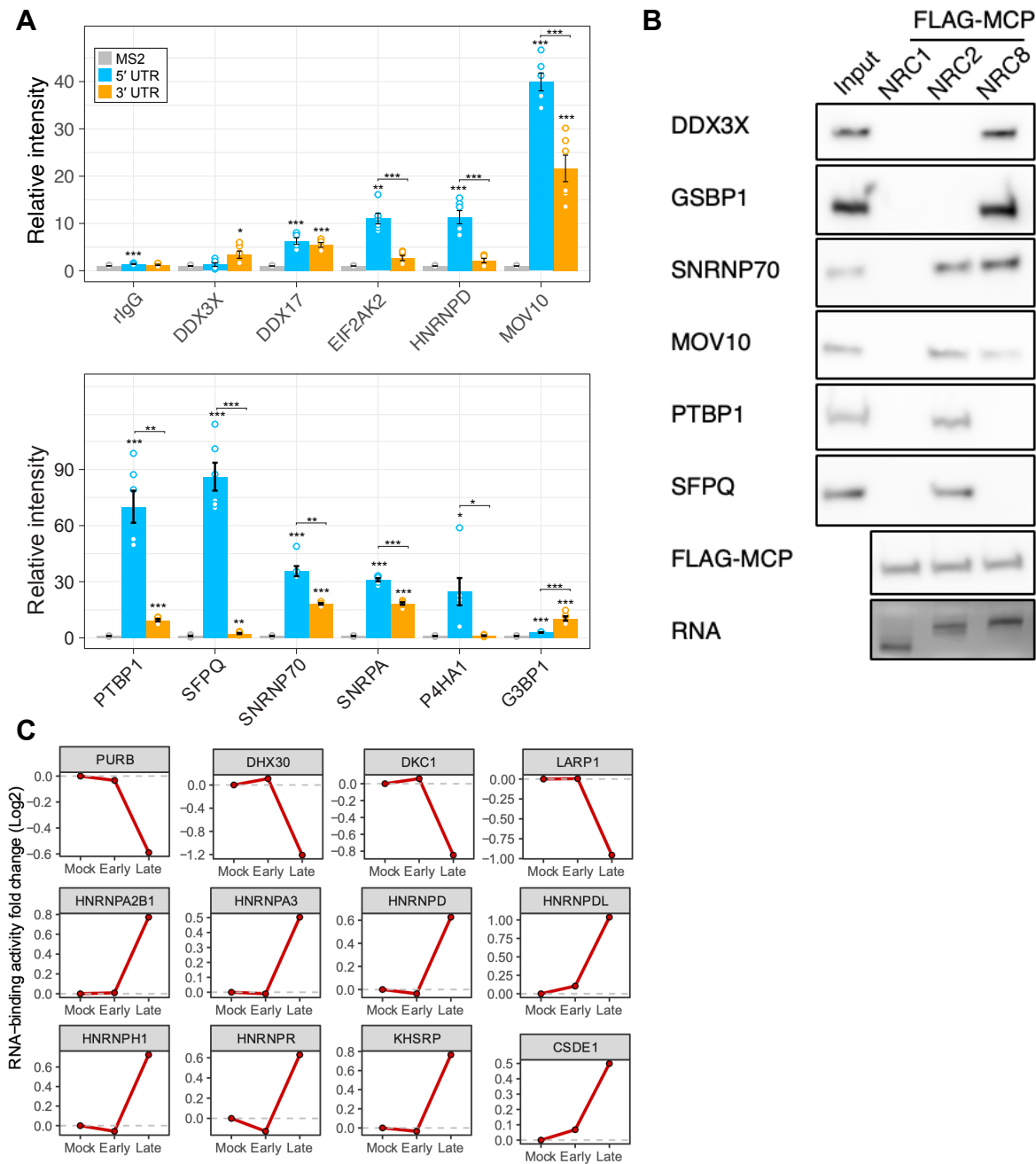

Supplement: Fig. S5 — Functional characterizations of the core SARS-CoV-2 ncrRNA interactome. (A) RIP experiments validating the interactions between host proteins and SARS-CoV-2 5' UTR or 3' UTR. P values were calculated by the Wilcoxon test. *P< 0.05, **P < 0.01, ***P < 0.001. Ribbit IgG proteins (rIgG) were used as a negative control. (B) Western blot experiments validating the interactions between host proteins and SARS-CoV-2 5' UTR or 3' UTR. (C) Fold change of proteins in the core SARS-CoV-2 ncrRNA interactome overlaying with published temporal abundance data of RBP during SARS-CoV-2 infection. Early represents 8 hpi. [file msystems.00135-23-s0005.pdf]

Figure S6

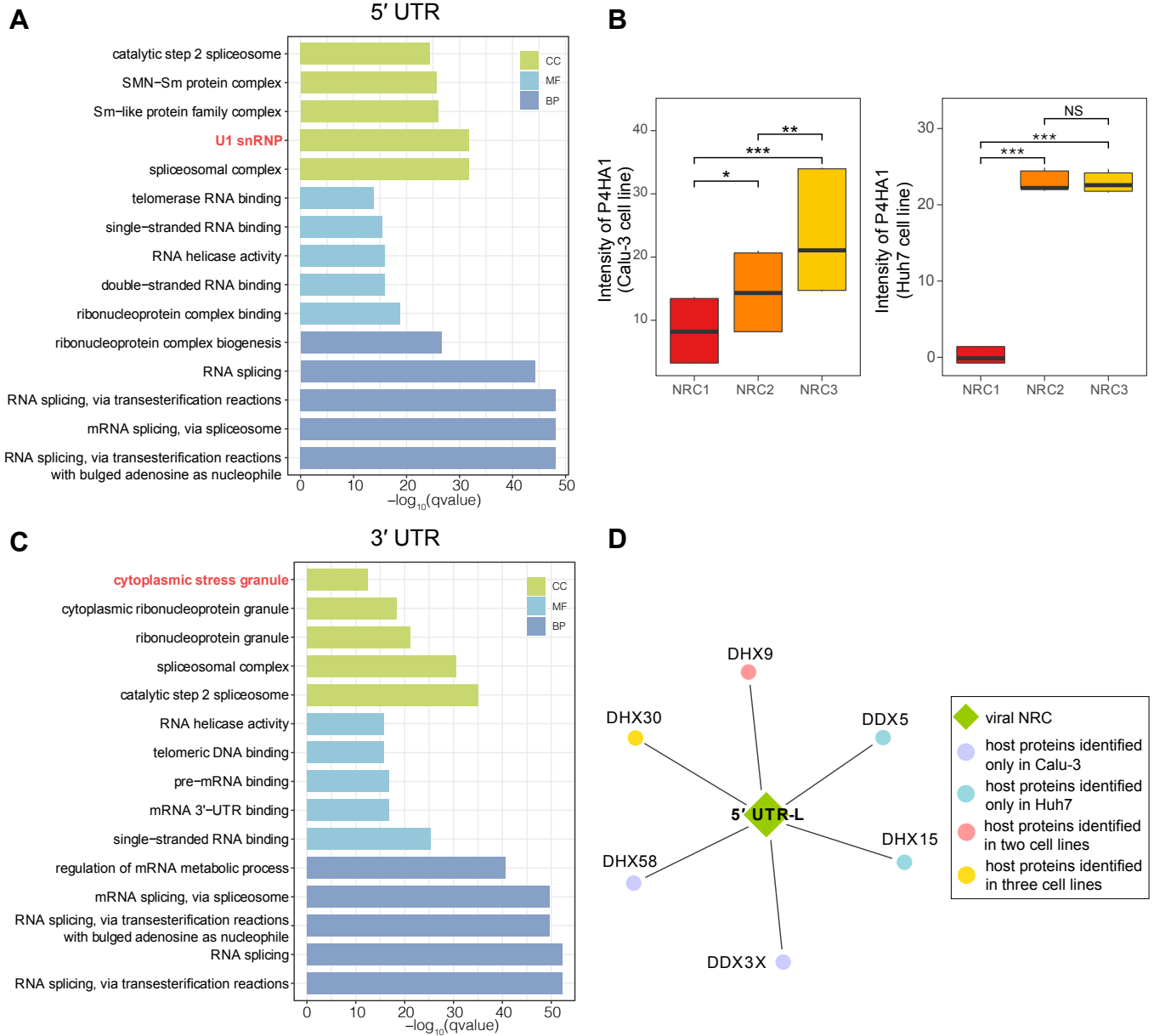

Supplement: Fig. S6 — The interactome of SARS-CoV-2 5' UTR, 3' UTR, and 5' UTR-L. (A) GO enrichment results revealing the major functions of the 5' UTR interactome. (B) The boxplot showing the intensity of P4HA1 in Calu-3 and Huh7 cells. P values were calculated by the Wilcoxon test. *P < 0.05, **P < 0.01, ***P < 0.001. (C) GO enrichment results revealing the major functions of the 3' UTR interactome. (D) Network revealing the interactions between DEAD/DEAH-box helicases and 5' UTR-L. Each node represents a host protein (closed circle) or viral ncrRNA (diamond). Colors are denoted in the box. [file msystems.00135-23-s0006.pdf]

Figure S7

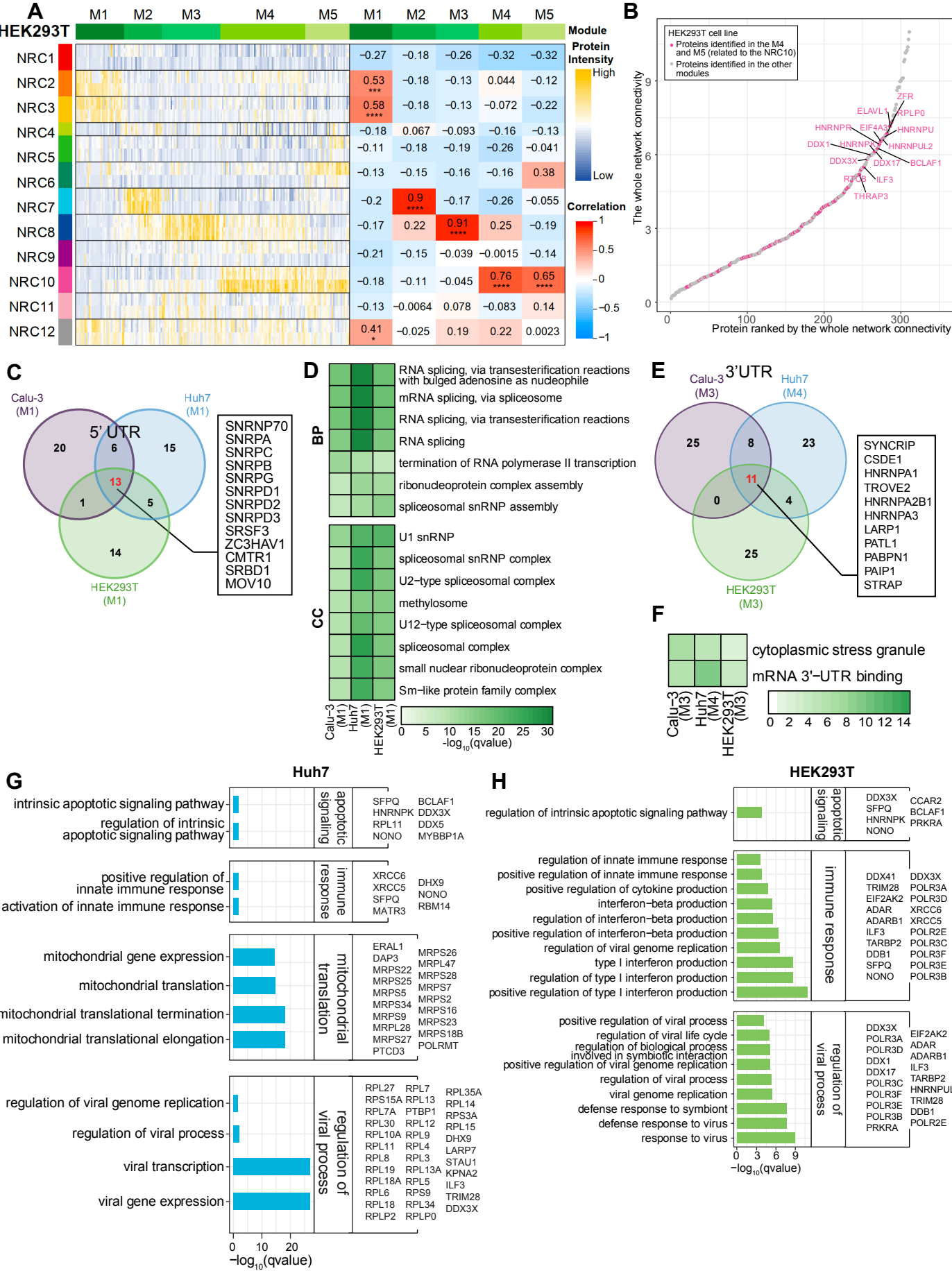

Supplement: Fig. S7 — ncrRNA-related modules identified by WGCNA. (A) Heatmap and table showing that modules were strongly related to different NRCs based on the interactions between host proteins and NRCs in the HEK293T cell line. Heatmap shows the intensity patterns of host proteins in different ncrRNA interactomes. The table shows the correlations between different modules and NRCs. P values were adjusted by the Bonferroni method. *P < 0.05, **P < 0.01, ***P < 0.001, ****P < 0.0001. See also Table S1. (B) Plots showing that the whole network connectivity of proteins identified in the modules correlated with the NRC10 in HEK293T cells. The top 15 proteins were highlighted in color pink. (C) Venn diagram of 5' UTR-related modules in Calu-3, Huh7, and HEK293T cells. (D) GO enrichment results reveal the major functions of the 5' UTR-related modules. (E) Venn diagram of 3' UTR-related modules in Calu-3, Huh7, and HEK293T cells. (F) GO enrichment results reveal the major functions of the 3' UTR-related modules. (G) GO enrichment analysis of NRC10 interactomes in Huh7 cells. (H) GO enrichment analysis of NRC10 interactomes in HEK293T cells. [file msystems.00135-23-s0007.pdf]

Figure S8

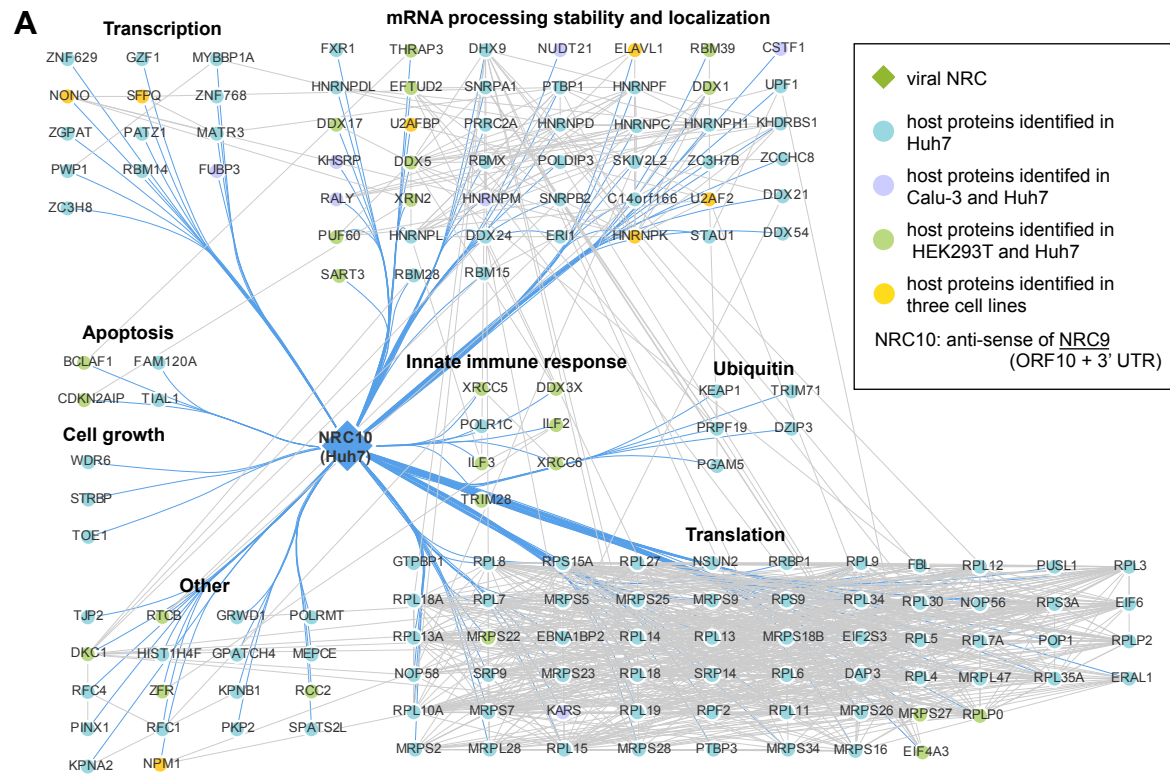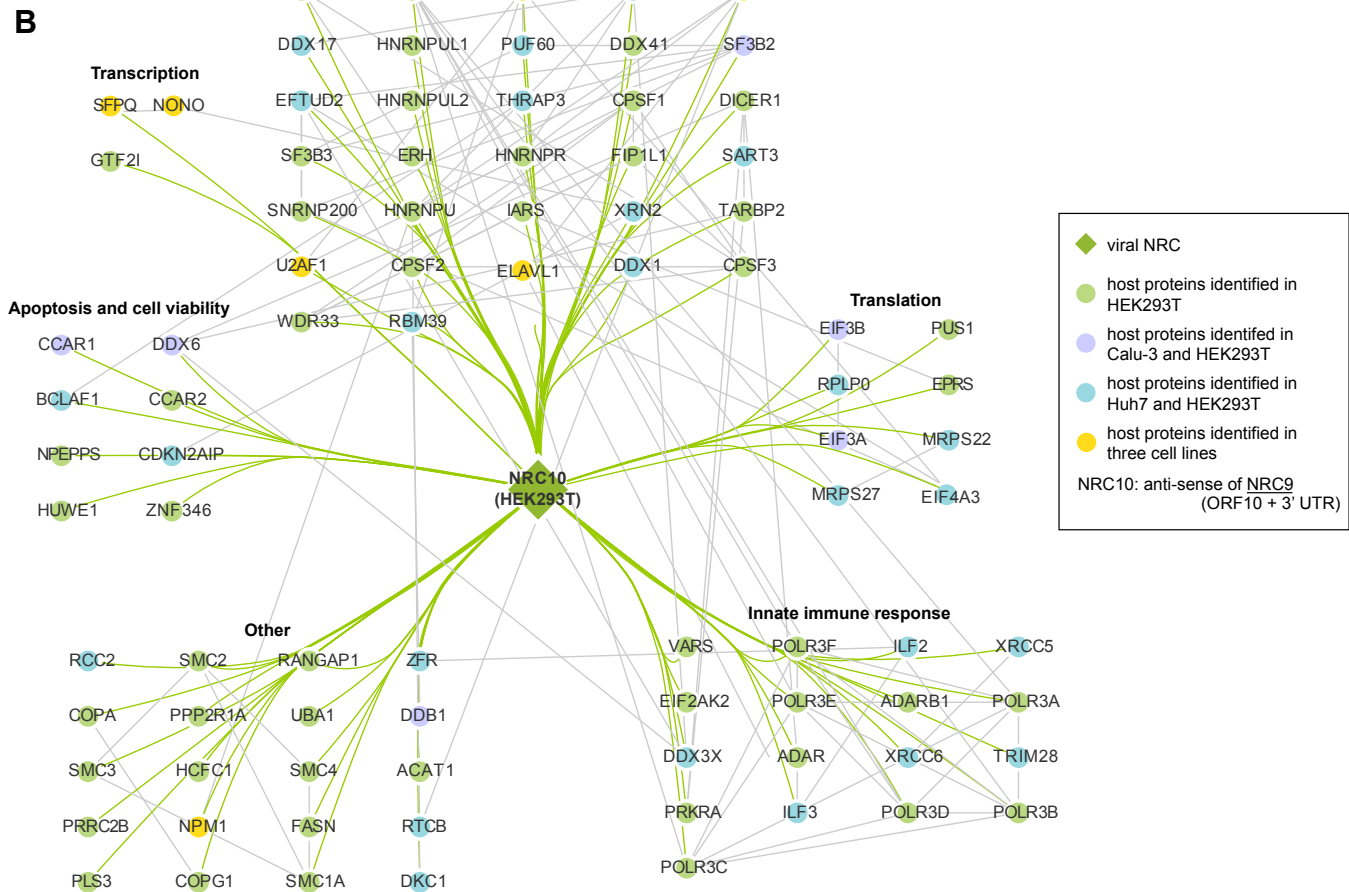

Supplement: Fig. S8 — Networks of NRC10 interactomes in Huh7 (A) and HEK293T (B) cells. Each node represents a host protein (closed circle) or viral ncrRNA (diamond). Blue or green edges indicate the interactions between viral ncrRNAs and host proteins. Gray edges denote the interactions in host proteins. Colors are denoted in the box. [file msystems.00135-23-s0008.pdf]
